# Advancements in Ultrasound Gel Pad Technologies: Enhancing Diagnostic Precision, Procedural Efficiency, and Therapeutic Applications

**DOI:** 10.3390/gels12050447

**Published:** 2026-05-19

**Authors:** Khair Ul Wara, Muhammad Hasan Masrur, Rana Talha Khalid, Hadiya Malik, Komal Tariq, Abdul Alber, Sang-Eun Song, Jawad Hussain, Saad Abdullah

**Affiliations:** 1 Department of Biomedical Engineering, Riphah International University, Lahore 54660, Pakistan; khairulwaraaa@gmail.com (K.U.W.); talha.k.rajpoot@gmail.com (R.T.K.); hadiya.malik@riphah.edu.pk (H.M.); t.komaaltariq@gmail.com (K.T.); abdulalber55@gmail.com (A.A.); jawad.hussain@riphah.edu.pk (J.H.); 2 Department of Biomedical Engineering, University of Glasgow, Glasgow G12 8QQ, UK; m.masrur.1@research.gla.ac.uk; 3 Department of Mechanical Engineering, University of Akron, Akron, OH 44325, USA; ssong@uakron.edu; 4 Department of Computer Science & Engineering, Mälardalen University, P.O. Box 883, 721 26 Västerås, Sweden

**Keywords:** ultrasound gel pads, acoustic coupling, hydrogel materials, drug delivery, gel pad fabrications, biocompatible and sustainable gel pads, 3D-printed pads, diagnostic imaging, therapeutic ultrasound, infection control

## Abstract

Ultrasound coupling technology is pivotal to ensuring high-quality diagnostic imaging, yet conventional water-based gels face persistent challenges, including acoustic impedance mismatch, air-bubble formation, dehydration, messiness, and cross-contamination risks. This review presents a comprehensive analysis of the evolution, materials science, and clinical performance of ultrasound gel pads, an advanced alternative engineered for superior acoustic transmission, hygiene, and patient comfort. Historical progression from early coupling agents to modern polymeric and hydrogel-based pads is traced, highlighting breakthroughs such as bilayer hydrogels, nanocomposite reinforcements, metamaterial-inspired designs, and patient-specific 3D-printed pads. Comparative evaluations demonstrate that gel pads, particularly those integrating nanotechnology, rival but often outperform traditional gels in transmission efficiency, near-field resolution, and adaptability to complex anatomical surfaces, while offering reusability and reduced environmental impact. For instance, solid gel pads achieved 92.3% stone disintegration, compared with 45.5% for semi-liquid gel, in ESWL phantom studies (*p* < 0.001). Materials, including polyacrylamide, silicone, and advanced hydrogels, are analyzed for mechanical properties, biocompatibility, and sustainability, with emphasis on biodegradable and locally sourced alternatives. Manufacturing innovations ranging from continuous casting to additive manufacturing enable customization, functional integration, and scalable production, although cost, supply chain stability, and regulatory compliance remain critical barriers. By uniting advances in materials engineering, nanotechnology, and precision manufacturing, ultrasound gel pads have demonstrated strong potential to advance coupling media for diagnostic, therapeutic, and wearable ultrasound applications, enabling higher diagnostic accuracy, streamlined workflows, and patient-centered care across diverse clinical and resource-limited settings.

## 1. Introduction

Ultrasound imaging plays a vital role in modern diagnostics, with over 120 million procedures performed annually in the US alone, and a global medical ultrasound equipment market projected to reach $13.2 billion by 2032 [1,2]. However, water-based gels used for acoustic coupling rapidly dehydrate, requiring reapplication every 15–20 min for prolonged sessions. They have also been linked to documented outbreaks of infection with *Pseudomonas aeruginosa*, *Burkholderia cepaciae*, *Escherichia coli*, and *Staphylococcus aureus*. Preservatives such as phthalates or parabens may produce allergic contact dermatitis in approximately 1–2% of patients [3,4,5]. These issues can reduce image quality, prolong procedures, and increase infection risk, especially in resource-limited settings [6,7]. Globally, these challenges are amplified in low- and middle-income countries (LMICs), where the World Health Organization estimates that over 4 billion people lack access to basic medical diagnostic imaging. A consistently identified barrier to sustainable ultrasound use in these resource-constrained settings is the prohibitive cost and scarce availability of commercial coupling media. Therefore, there is an urgent need for advanced, low-cost options such as ultrasound gel pads to improve diagnostic access, safety, efficiency, and accuracy [8,9].

Ultrasound gel pads serve as acoustic couplants to facilitate efficient transmission of high-frequency sound waves from the transducer into soft tissue. The primary physical principle governing this process is acoustic impedance matching. The piezoelectric transducer (typically lead zirconate titanate, acoustic impedance Z ≈ 30–35 MRayl) and human soft tissue (Z ≈ 1.5 MRayl) exhibit a large mismatch, while air (Z ≈ 0.0004 MRayl) causes nearly total reflection (>99.9%). A gel pad with intermediate acoustic impedance (1.5–1.7 MRayl) minimizes reflection losses and maximizes energy transmission (>90%), directly improving image quality by reducing dead zones and near-field artifacts. Attenuation within the coupling layer must remain below 0.2 dB/cm at 5 MHz; higher values degrade signal-to-noise ratio and limit penetration depth. Phantom studies have shown that gel pads with optimized impedance and low attenuation yield 15–35% higher near-field resolution and significantly lower air-trapping artifacts compared with traditional gels [10]. Ultrasound imaging is a foundational technology in modern medicine, with the coupling medium—most often traditional ultrasound gel—playing a critical role in the quality and safety of diagnostic procedures. The primary function of these gels is to eliminate air between the transducer and the skin, as air’s low acoustic impedance severely impedes ultrasound wave transmission. Afzal et al. [11] demonstrated that a well-formulated gel with an acoustic impedance of 1.5 MRayl enables complete transmission of ultrasound waves, resulting in high-quality images, while also noting that commercial gels can be prohibitively expensive in resource-limited settings, restricting access to essential diagnostics. The cost and availability of commercial gels remain a global challenge, particularly in low-resource environments, as highlighted by Riguzzi et al. [12], who found that homemade cornstarch-based gels produced images rated as accurate in 70.6% of scans, compared to 65.2% for commercial gels, with no significant difference in image detail, resolution, or quality. The physicochemical properties of traditional gels, such as clarity, homogeneity, pH, viscosity, and spreadability, are critical for their performance. Al-Nima et al. [4] reported that a carbopol-based gel with a pH of 6.8, close to skin pH, showed no significant difference in density, viscosity, or spreadability compared to commercial gels, and did not cause skin irritation in animal or human tests. However, the use of certain preservatives and thickening agents, such as isothiazolinones, methylisothiazolinone, and phenoxyethanol, has been associated with allergic contact dermatitis, raising concerns about the safety of these additives in clinical practice [13,14,15,16].

Microbial contamination is a significant risk associated with traditional ultrasound gels. Oleszkowicz et al. [3] described outbreaks of *Pseudomonas aeruginosa*, *Escherichia coli*, and *Staphylococcus aureus* infections linked to contaminated gels, with one series involving 16 patients who developed respiratory tract infection after cardiovascular surgery. Molecular typing confirmed the gel as the source, and the outbreak was halted by switching to single-use sterile gel. The lack of intrinsic antimicrobial properties in many gels, despite claims of bacteriostatic activity, further exacerbates this risk, as shown by studies where both opened and unopened bottles of gel supported bacterial growth. In addition to infection risks, the technical limitations of traditional gels can impact diagnostic accuracy. Kim et al. [17] found that traditional gels require reapplication every 15 min due to drying, which leads to impedance mismatch and reduced imaging resolution. Their study showed that a soft solid gel outperformed traditional gel in grayscale, dead zone, vertical, and horizontal imaging parameters, with values of 105.64 mm, 34.48 mm, 141.1 mm, and 102.8 mm, respectively, compared to 93.79 mm, 45.32 mm, 103.13 mm, and 83.86 mm for traditional gel. The need for frequent reapplication not only disrupts workflow but also increases the risk of introducing air bubbles, which can cause artifacts such as shadowing and reverberation, potentially leading to misdiagnosis.

Yi et al. [18] addressed the challenges of intraoral ultrasound imaging, where commercial gel pads like Aquaflex face issues of low stability in water, poor mechanical properties, and potential cytotoxicity. Their research on polyacrylamide/sodium alginate double-network hydrogels demonstrated improved stability, mechanical strength, and biocompatibility, while providing similar image quality to commercial pads and lower cytotoxicity to both cancer and fibroblast cells. This highlights the potential for advanced hydrogel formulations to overcome some of the inherent limitations of traditional gels, particularly in specialized applications.

Following the discussion of market advantages and limitations of conventional liquid ultrasound gels (e.g., messiness, patient discomfort, and residue), gel pads have emerged as a superior alternative. Compared with traditional liquid coupling gels, solid or semi-solid gel pads provide comparable or superior image quality while offering markedly improved patient comfort, no adherence to body hair, easy removal without residue, and better conformability to high-curvature anatomical sites. These pads also demonstrate enhanced mechanical stability and handling during prolonged or intraoperative procedures. Recent clinical evaluations confirm that gel pads yield a significantly higher contrast-to-noise ratio and signal-to-noise ratio at tissue interfaces and receive higher volunteer satisfaction scores than liquid gels, with no compromise in diagnostic utility [19,20].

The use of ultrasound gel extends beyond diagnostic imaging to therapeutic procedures. Ren et al. [21] investigated the application of gel to mitigate skin burns during microwave ablation, finding that a 4 cm thick gel layer at an 8 cm applicator insertion depth reduced temperature elevations from 39.9 °C to 23.2 °C (*p* < 0.001), demonstrating a significant protective effect. This underscores the versatility of ultrasound gel in enhancing patient safety across a range of medical interventions.

In the context of food science, ultrasound-assisted processing has been shown to improve the gelling properties of proteins. The rationale for this review is the growing need for advanced ultrasound coupling media that address the limitations of conventional gels in image quality, procedural efficiency, and patient safety. This review provides a comprehensive overview of gel pad technologies, tracing their historical evolution; the underlying materials science of polymers, hydrogels, and nanocomposites; and key fabrication methods including casting, injection molding, and 3D printing. It also examines clinical applications, biocompatibility, sustainability, and customization, while addressing barriers such as cost and scalability. Overall, the review aims to guide future innovation in gel pad technologies to deliver more effective, efficient, and responsible solutions for patient care across diverse clinical and resource-limited settings.

## 2. Review Methodology

This narrative literature review was conducted in accordance with the Preferred Reporting Items for Systematic Reviews and Meta-Analyses (PRISMA) guidelines to ensure methodological transparency, reproducibility, and scientific rigor. A comprehensive search was performed in four major databases—PubMed, Scopus, ScienceDirect, and IEEE Xplore—covering the period from January 2000 to August 2025. The search strategy combined controlled vocabulary (MeSH terms where applicable) and free-text keywords using Boolean operators. Core search terms included “ultrasound gel,” “gel pad,” “standoff pad,” “coupling medium,” “acoustic impedance,” “attenuation,” “polymer gel,” “hydrogel,” “nanocomposite gel,” “3D-printed pad,” and “infection control,” with additional targeted combinations for therapeutic ultrasound, drug delivery, and sustainability aspects.

A total of 1872 records were retrieved. After automated removal of 527 duplicates, 1345 unique records remained. Two independent reviewers (K.U.W. and M.H.M.) performed title-and-abstract screening using Rayyan software (accessed January 2026), applying the preliminary relevance filter. This stage excluded 1033 records, leaving 312 articles for full-text assessment. Full-text articles were then evaluated against the following pre-defined inclusion and exclusion criteria.

The inclusion criteria comprised studies reporting quantitative acoustic or mechanical assessments of transducer coupling materials, including impedance, attenuation, and transmission efficiency. Comparative studies evaluating conventional gels against polymeric or solid gel pads, as well as advanced hydrogel formulations, were also included. Clinical and user-based studies reporting image quality, workflow efficiency, hygiene, infection control, patient comfort, or procedural outcomes were considered eligible. In addition, studies describing the design, fabrication, or manufacturing of novel coupling media, including approaches based on 3D printing, nanotechnology, or sustainable materials, were included. The exclusion criteria comprised non-peer-reviewed articles, conference abstracts, editorials, and preprints lacking a complete methodology. Studies without quantitative data on acoustic or mechanical performance, or without relevant clinical outcomes, were excluded. Articles focused solely on non-medical ultrasonics, such as food processing, industrial cleaning, or other non-biological applications, were also excluded. Duplicate publications and studies with insufficient methodological detail for reliable data extraction were not considered.

Disagreements between the two reviewers were resolved by consensus discussion with a third reviewer (S.A.) when necessary. Ultimately, 178 studies met all inclusion criteria and were included in the qualitative synthesis. The full reference list contains 313 citations, including background literature, methodological references, and supporting studies cited throughout the review for contextual and historical purposes.

**Figure 1:**
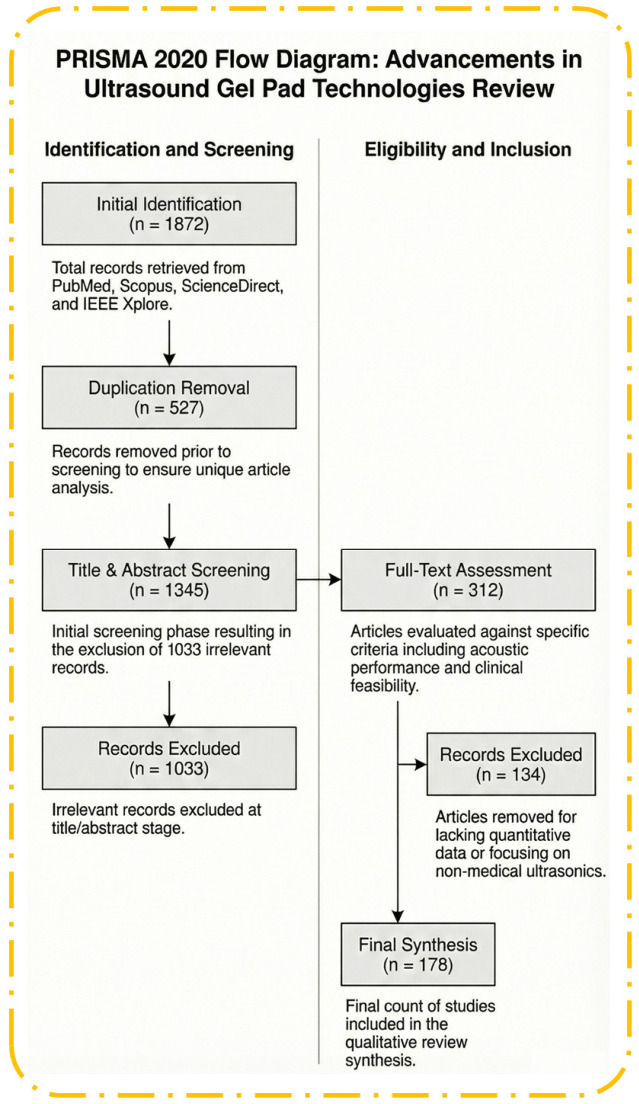
PRISMA 2020 flow diagram illustrating the study selection process (adapted from Page et al. [22]).

The study selection process followed the PRISMA 2020 statement [22], and the results are summarized in Figure 1.

Data extraction was performed independently by two reviewers using a standardized form that captured key parameters: acoustic impedance (MRayl), attenuation coefficient (dB/cm/MHz), coupling efficiency, dehydration rate, biocompatibility metrics (cytotoxicity, irritation), mechanical properties (tensile strength, elongation), manufacturing method, clinical setting, and sustainability indicators. Thematic synthesis grouped findings into acoustic performance, material stability and biocompatibility, infection control, manufacturing innovations, clinical applications, therapeutic uses, sustainability, and future directions. No statistical meta-analysis was performed because of heterogeneity in study designs and outcome measures; results are presented narratively with quantitative data reported where available.

This structured, transparent, and reproducible methodology ensures that the review is both comprehensive and verifiable, addressing previous limitations noted in the field.

## 3. Historical Evolution of Ultrasound Coupling Media

Materials science dynamics have certainly had an impact on the evolution of ultrasound couplet agents concerning clinical requirements and technological progress. The multifunctional agents currently being used in diagnostic ultrasound were developed in sequence with basic aqueous solutions in the early 1950s, when diagnostic ultrasound was becoming a reality. First, there was the use of water, mineral oils, and glycerin during the 1950s–1970s. These agents were simple to use; however, they had low acoustic impedance, which was equivalent to that of water, and a poor image was obtained just a matter of 10 min to 20 min after application [23,24,25]. Details of acoustic impedance matching, air-bubble formation, and dehydration are discussed in Section 1. The introduction of water-based gels in the late 1960s and 1970s, such as carbomer and methylcellulose gels, marked a significant improvement. These gels-maintained coupling for 45–60 min; attenuation reduced to 0.5 dB/cm at 5 MHz [26,27,28,29]. However, they still dried out over time, especially under the heat of transducers, and required reapplication for procedures exceeding one hour. Hydrogels, introduced in the 1980s, offered water retention rates above 90% by weight and could maintain their structure for up to 8–24 h, with degradation primarily due to slow dehydration [30,31]. Cellulose-based gels, which emerged in the 1990s, provided additional benefits of biodegradability and non-toxicity, with mechanical stability for up to 24 h in laboratory conditions [32].

The commercialization of semi-solid, pre-formed gel pads in the mid-1990s represented a significant breakthrough. These pads, typically 5–10 mm thick and composed of polyacrylamide or silicone hydrogels, could be used for up to 4–12 h without significant degradation [33,34]. Quantitative studies showed that these pads reduced acoustic attenuation to less than 0.2 dB/cm at 5 MHz and maintained coupling integrity for procedures lasting up to 12 h [34]. Moldable gel pads, introduced in the early 2000s, allowed for custom shaping to fit irregular anatomical surfaces, further reducing pressure artifacts and improving patient comfort. Recent years have seen the integration of nanotechnology and advanced polymers into gel pad formulations. These materials can maintain structural integrity for up to 72 h in sealed packaging and 24–48 h in clinical use, with acoustic attenuation as low as 0.15 dB/cm at 5 MHz [35,36]. 3D-printed gel pads, first reported in 2018, can be tailored to patient anatomy and maintain performance for up to 48 h [37]. Non-Newtonian fluid-based gels, developed for wearable ultrasound, exhibit a dehydration rate of just 32% over 24 h and an acoustic attenuation of 0.328 dB/cm/MHz [38].

El-Sherif et al. [39] investigated the degradation of poly(lactic-co-glycolic acid) (PLGA) microcapsules used as ultrasound contrast agents. They found that the degradation rate was closely related to capsule morphology and acoustic efficiency, with more efficient capsules degrading faster, especially when insonated at their optimal frequency (5 or 10 MHz). Quantitatively, the most efficient capsules (maximum enhancement of 25 dB at 5 MHz with 0.004 mg/mL) degraded significantly faster than less efficient ones (requiring 0.6 mg/mL for the same enhancement). More glycolic acid was released at an early point, reflecting the greater hydrophilicity and degradation rate of glycolic acid repeat units. Fagan et al. [40] demonstrated that coupling ultrasound with activated persulfate significantly enhances the degradation of polycyclic aromatic hydrocarbons (PAHs) in aqueous solutions. At 20 °C, the observed rate constants for ultrasonically activated persulfate (US-PS) correlated strongly with diffusion coefficients, indicating that PAH molecules diffuse to the bubble–water interface before reacting with sulfate radicals. The synergy index for PAHs with fast diffusion coefficients was excellent at 20 °C, and the maximum synergy for fluoranthene was observed at 30 °C. However, synergy decreased at higher temperatures due to increased radical–radical recombination. These findings highlight the importance of temperature and physicochemical parameters in optimizing ultrasound-assisted degradation processes.

Neppolian et al. [41] and Kritikos et al. [42] discussed the sonolytic and photocatalytic degradation of organic contaminants, and they both showed that ultrasound irradiation, particularly in the presence of an oxidizing agent such as persulfate or Fenton reagent, could degrade methyl tert-butyl ether (MTBE) over 95% and decolorize dyes, respectively, in less than 60 min. The degradation rate rose as ultrasonic power and temperature rose, and the presence of oxidizing agents further improved the rate of degradation. These studies give quantitative data to support the usefulness of ultrasound-coupled degradation both in the environment and in the biomedical setting. Siddique et al. [43] examined the decomposition of Reactive Blue 19 dye through photolysis, photocatalysis, and sonophotocatalysis. They discovered that the level of degradation was higher as the pH fell, the initial concentration of dye dropped, and the catalyst load and ultrasonic power were increased. It was found by the kinetic study that the degradation obeyed first-order reaction kinetics, and the interaction between ultrasound and photocatalysis was more effective than either of the two processes because of the increased availability of reactive hydroxyl radicals, or the number of catalyst surfaces. While the historical progression from water-based gels to modern hydrogel pads clearly demonstrates improved acoustic performance, many early studies lack long-term clinical validation and rely on phantom models that do not fully replicate real-tissue variability. Comparative data remain limited, with only a few head-to-head trials against commercial gels, underscoring the need for larger, multi-center studies before claiming broad superiority [44].

Table 1 draws attention to the evolution of ultrasound technology through the historical perspective of a sequence of transformative events that have influenced the present role of technology in the field of medicine and industry. Pioneers in medical ultrasound during the 1930s and 1940s, such as Theodore and Friedrich Dussik, laid the foundation for medical ultrasound by attempting to diagnose brain tumors. However, it was not until the 1970s that technological advancements made ultrasound a practical and widely used diagnostic tool [45]. The replacement of bulky, massive machines that entailed the patient immersion tanks in the 1950s with portable and handheld devices in the 2000s significantly increased accessibility and clinical usability, enabling real-time imaging in a variety of clinical specialties. Enhanced image quality, versatility, and miniaturization of the device were also achieved by the introduction of specialized probes, multiple imaging modes (A, B, M, D), and substitution of conventional piezoelectric crystals with capacitive micromachined ultrasound transducers (CMUTs) [46,47].

Ultrasound diagnostic power was enhanced in the late 20th century by the development of microbubble contrast agents, which made evaluating microvascular blood flow possible, as well as increased the use of ultrasound in cardiology and radiology [48]. The development in the materials of transducers and digital signal processors, as well as the development of high-frequency and dual frequency probes, has resulted in superior spatial and contrast resolution and has found use in clinical and research studies, especially in oncology and cardiovascular disease [49,50]. A combination of Doppler, elastography, and contrast-enhanced imaging has expanded the clinical span of ultrasound. Simultaneously, real-time high-resolution imaging and even AI-assisted ones were made possible by the miniaturization of electronics and the introduction of software-based systems [51,52,53].

**Figure 2:**
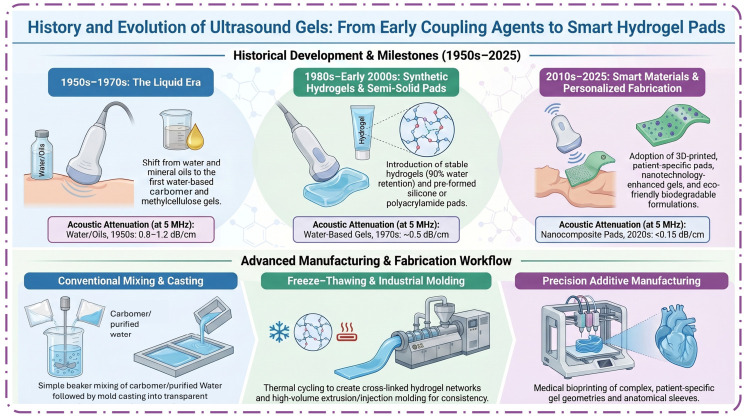
Timeline and technological evolution of ultrasound gel formulations and manufacturing techniques (1950s–2025). The blue, green, and purple panels represent successive developmental stages: the liquid era (1950s–1970s), synthetic hydrogels and semi-solid pads (1980s–early 2000s), and smart materials with personalized fabrication approaches (2010s–2025), respectively. Illustrated icons depict representative ultrasound probes, coupling materials, hydrogel network structures, freeze–thaw processing, industrial molding systems, and additive manufacturing/3D bioprinting technologies. Acoustic attenuation values shown in each stage indicate representative performance trends at 5 MHz. The lower panel summarizes major fabrication approaches, including conventional mixing and casting, freeze–thaw and industrial molding methods, and precision additive manufacturing for patient-specific gel structures.

In addition to medicine, ultrasound has been used extensively in the oil and gas, food processing, and manufacturing sectors as it is effective and versatile when it comes to non-destructive testing and optimization of processes [54,55]. Portable devices, training expansion, and the addition of AI have caused the standardization of ultrasound and have made point-of-care ultrasound and remote diagnostics more accessible than ever before [56,57]. All these progressive developments have made ultrasound a fundamental pillar of contemporary diagnostics and treatment, and these developments have been progressively growing with time. Figure 2 represents how ultrasound coupling agents were developed in the early water media and water immersion methods of the 1950s to highly developed hydrogel formulations, semi-solid pads, nanotechnology-enhanced gels, and 3D-printed gel pads by the year 2025. The timeline illustrates a clear progression from passive water-based media to active, stimuli-responsive hydrogel designs. However, clinical translation of recent metamaterial and 3D-printed pads remains limited by scalability and cost, highlighting the gap between technological innovation and routine clinical adoption. Major milestones include the introduction of water-based gels containing carbomer and methylcellulose in the 1960s–1970s, cellulose-based gels in the 1980s–1990s, moldable gel pads in the mid-1990s, and non-Newtonian gels with enhanced acoustic properties in the late 2010s. The manufacturing technologies developed from basic mixing and freeze–thawto mold casting, extrusion/injection molding, and 3D printing, and the trends of manufacturing in the future are eco-friendly and biodegradable formulation and improved acoustic performance.

**Table 1:** Key historical developments and their impact on ultrasound technologies.

No.	Reference	Key Historical Development	Impact on Ultrasound Technologies
**1.**	[45]	Early brain tumor diagnosis attempts (1930s–40s); evolution to portable, user-friendly machines	Established ultrasound as a significant diagnostic tool; improved accessibility and image quality
**2.**	[47]	Transition from immersion tanks (1950s) to handheld devices (2000s); probe variety	Enhanced portability, real-time imaging, and diagnostic versatility
**3.**	[48]	Development of microbubble contrast agents	Enabled assessment of microvascular blood flow; expanded diagnostic applications.
**4.**	[49]	Rise of high-frequency ultrasound (10–60 MHz); dual-frequency and multimodality probes	Improved resolution for research and clinical imaging, especially in cancer and cardiovascular disease
**5.**	[50]	Advances in transducer materials, digital processing, and microbubble agents	Improved spatial/contrast resolution; enabled functional and quantitative studies
**6.**	[51]	Ultrasound in the oil/gas industry: field and lab research	Demonstrated ultrasound versatility beyond medicine; improved industrial processes
**7.**	[52]	Introduction of Doppler, elastography, and contrast-enhanced ultrasound	Broadened clinical applications and improved tissue characterization
**8.**	[54]	Ultrasound in food processing	Revolutionized the food industry with efficient, non-destructive processing
**9.**	[55]	Non-linear imaging, Doppler modes, 3D/4D, elastography	Improved tissue contrast, blood flow imaging, and integration with digital systems
**10.**	[56]	3D/4D imaging; AI; elastography	Enhanced diagnostic accuracy and personalized medicine
**11.**	[57]	Democratization: portable/handheld devices, AI, expanded training	Increased global accessibility and point-of-care use
**12.**	[58]	Langevin’s quartz transducer (1918); sonar for military, then medical use	Foundation for piezoelectric transducers; spurred diagnostic and therapeutic ultrasound
**13.**	[59]	Miniaturization of electronics; digital beamformers; 3D/4D imaging	High-resolution, real-time imaging; expanded musculoskeletal and interventional uses
**14.**	[60]	Early 20th-century exploration; shift from therapy to diagnosis	Set the stage for safe, practical diagnostic ultrasound
**15.**	[61]	Military sonar to post-WWII medical/industrial ultrasound	Paved the way for peaceful, widespread ultrasound applications
**16.**	[62]	Focused ultrasound in ophthalmology (1950s onward)	Enabled high-resolution eye imaging and novel therapies

## 4. Conventional Ultrasound Coupling Media

A coupling medium of ultrasound, such as air that exists between the transducer and the skin, is indispensable in ensuring that sound waves are transmitted effectively and that images will be of high quality. Traditional water-based gels are still the most utilized; however, in recent times, there has been an extension to polymeric hydrogels, plant-based alternatives, and non-Newtonian formulations. Afzal et al. [11] have created carbopol-based gels that were stable and gave equal image clarity compared to commercial gel; their conductivity values were 2.10 mS/cm, and commercial formulations were 0.70 mS/cm, and microbial contamination was absent after 30 days. Plant-based media have also been demonstrated to be of clinical interest. Prachasilchai et al. [63] reported that rice gel, which was tested on 100 patients, had the same clarity and better echogenicity than commercial gel, whereas Neto et al. [64] reported that Aloe vera gel had the same or better imaging quality because of its lower electrical resistance and higher free-ion content. Equally, cassava starch gels used in liver imaging did not show any notable difference in image quality to commercial agents [65]. More sophisticated material, like non-Newtonian acoustic gels, has been biased to be wearable, with lateral resolutions 35% better and dehydration at 32% after 72 h in the 4.60–10.60 MHz range [66]. Taken together, all these findings reflect a trend towards biocompatible and application-targeted alternatives to the conventional coupling gels. Regardless of benefits, non-Newtonian gels add 15–20% to the cost in wearable devices [37].

### 4.1. Gel Pads vs. Traditional Gels

In medicine, gels and gel pads find their use in ultrasound imaging, extracorporeal shock wave lithotripsy (ESWL), and surgical positioning, among other applications, and material properties are important in determining the imaging quality of the former, effectiveness of treatment by the former, and patient safety of the latter. Gels, which are usually semi-liquids, offer acoustic coupling by reducing the number of air gaps between the skin and medical apparatus but their effectiveness can be influenced by uneven application and anything that is left in the air. Gel pads are manufactured solid or semi-solid coupling materials designed to improve acoustic transmission, reduce the force applied to the patient, enhance reusability, and facilitate easier cleaning compared with conventional gels, which may have variable quality and create mess during use. Some systematic quantitative comparisons between such materials, e.g., disintegration rates of stone in ESWL, pressure ulcer rates in long surgeries and release of water in controlled applications, were carried out in a few studies. These comparisons consider the potential benefits in clinical outcomes and the efficiency in procedures afforded by gel pads to the medical professionals and researchers. The comparison of gels and gel pads reveals critical factors in imaging quality, anatomical use and reusability.

Wang et al. [67] evaluated a proprietary solid gel pad (icPad) vs. classical semi-liquid gel in a phantom model for extracorporeal shock wave lithotripsy (ESWL). The icPad is manufactured of polyacrylamide in 4 mm and 8 mm thicknesses. An identifiably higher rate of stone disintegration was measured after 200 shock waves at energy level 2:4 mm icPad was 92.3%, 8 mm icPad was 85.0%, and semi-liquid gel was only 45.5% (*p* < 0.001). Furthermore, the number of shocks required for complete stone disintegration was lower with the icPad (242.0 ± 13.8 for 4 mm, 248.7 ± 6.3 for 8 mm) than with the semi-liquid gel (351.0 ± 54.6, *p* = 0.011). Quantitative imaging also revealed that the area of trapped air pockets was much smaller with the icPad (50.3 ± 31.9 mm^2^) than with the gel (332.7 ± 91.2 mm^2^), indicating superior acoustic coupling and energy transmission. As detailed in Section 1, traditional gels are prone to air-bubble formation and impedance mismatch. Gel pads address these issues through their solid structure, as shown by the quantitative comparisons below. In the context of patient safety and clinical outcomes, Zeng et al. [68] used a large-scale retrospective cohort study to compare clinically traditional gel pads with fluidized positioners in regard to skin protection among 706 neurosurgical patients. The sample size after the propensity score matching was 394 fluid positioners and 312 gel pad patients. The pressure ulcer rates were found to be much lower in the fluidized positioner (3.7) than the gel pad group (7.8, *p* = 0.034). Also, the length of stay of the hospitals decreased in fluid positioner group (22.35 vs. 25.65 days, *p* < 0.001). It was also shown in pressure mapping that gel pads placed greater localized stress on the skin whereas fluidized positioners placed the pressure in a more balanced way and injury was less likely. This paper reveals that gel pads are indeed useful but other new products might provide better skin protection in long-lasting operations. Other major considerations on the strength of gel pads and gels are material characteristics and water release. Comparing waters released by different agar gel formulations and conventional pads, Sansonetti et al. [69] established that water released by paper pulp gel and sepiolite gel were 2–4 times greater than water released by agar gels. Adding agar by 1 percent water decreased the water release by 16.98–66.88 g, according to the substrate, and the incorporation of Japanese tissue paper further decreased water release by 18–76%. The presented quantitative results indicate that the composition and structure of gel pad can be optimized to direct the transfer of moisture that is essential in both medical and conservation practice. Voniatis et al. [70] joined the research on gel and liquid handrubs but the study did not focus on medicine, so the comparison of both types of handrubs was conducted among 340 participants, which gives appropriate data about handling and covering with gels. Both gel and liquid had substantial areas of the hands uncovered at a 1.5 mL application volume (7.0% and 5.8% respectively). Increasing the applied volume to 3 mL reduced the uncovered areas to less than 1.5% for both formats. Conventional gels performed better at higher volumes, whereas liquid formulations were more effective at lower volumes. These findings suggest that increasing the application volume of gel pads may improve coverage reliability and reduce spillage during clinical ultrasound procedures, supporting their use in applications requiring stable and consistent acoustic coupling.

**Figure 3:**
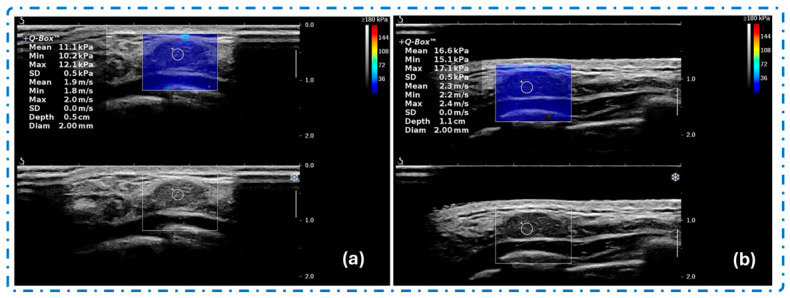
Comparative shear-wave elastography (SWE) ultrasound images demonstrating the effects of different acoustic coupling conditions on superficial tissue imaging. (**a**) SWE image acquired using conventional coupling conditions showing reduced uniformity and potential near-field artifacts. (**b**) SWE image acquired under improved coupling conditions demonstrating enhanced image clarity and more consistent acoustic coupling. Images in (**a**,**b**) are reprinted from Taylor & Francis under the Creative Commons Attribution 4.0 license [71].

Zhang et al. [71] investigated the effect of gel-assisted coupling conditions on the repeatability and diagnostic performance of shear wave elastography (SWE) for superficial breast lesions. As shown in Figure 3, the comparison between conventional coupling gel and improved coupling conditions demonstrates differences in image uniformity and near-field visualization. Conventional gel application may produce inconsistent acoustic coupling and localized artifacts due to uneven probe contact, particularly over superficial or irregular anatomical regions (Figure 3a). In contrast, optimized coupling conditions provide more stable probe positioning and improved acoustic transmission, resulting in clearer visualization and enhanced measurement consistency (Figure 3b). These findings suggest that improved coupling interfaces can enhance SWE image quality and diagnostic reliability for superficial tissue assessment [72].

### 4.2. Material Classes and Designs of Ultrasound Gel Pads

Ultrasound gel pads are engineered to optimize acoustic coupling (as detailed in Section 1), especially in situations where traditional gels may be less effective or practical. The following categories summarize the main types of ultrasound gel pads, highlighting their unique properties, clinical applications, and supporting quantitative data. Each section includes multiple examples to illustrate the diversity and innovation in this field.

Polyacrylamide/Alginate double-network (DN) hydrogel pads offer a significant improvement over traditional couplants like Aquaflex, which suffer from low water stability, weak mechanical strength, and cytotoxicity. Double-network (DN) hydrogels consist of two interpenetrating polymer networks with contrasting mechanical properties: a rigid, densely cross-linked first network that dissipates energy through sacrificial bond rupture and a soft, ductile second network that provides elasticity and recovery. This architecture yields hydrogels with exceptional toughness, stretchability, and fatigue resistance, making them particularly suitable for ultrasound gel pad applications [73]. PAM/Alginate DN hydrogels show superior water stability, higher friction for probe stability, and comparable imaging quality, while demonstrating lower cytotoxicity to fibroblast and cancer cells, making them safer for clinical intraoral and dental applications [18]. Wang et al. [74] have further enhanced DN hydrogel performance. Fiber-reinforced sodium alginate/PAM DN hydrogels achieved tensile strength of 0.83 MPa, stretchability of 4230%, and toughness of 15.7 MJ/m^3^, surpassing conventional hydrogels. Alginate-reinforced PAM/xanthan gum DN ionic hydrogels combine tensile strength up to 0.65 MPa, fracture strain 1800%, and excellent conductivity, enabling use in biomedical sensors and wearables [75]. Hybrid DN hydrogels with zwitterionic polymers reached an elastic modulus of 0.28 MPa and tensile strength of 0.69 MPa, with antifouling and biocompatibility suitable for scaffolds and implants [76]. Self-healing and adhesive DN hydrogels based on oxidized alginate/gelatin showed strain 2800%, stress 630 kPa, 79% self-healing efficiency, and conductivity 0.72 S/m, supporting use in flexible healthcare monitoring [77]. Copper–alginate PAM DN hydrogels delivered tensile strength 2.25 MPa and ionic conductivity 4.08 mS/cm [77], while graphene oxide- or chitosan-modified DN hydrogels maintained high conductivity even at low temperatures [78,79]. Additionally, nanocomposite DN hydrogels with silver nanoparticles exhibited fracture stress of 1255 kPa, modulus of 200 kPa, and antibacterial activity, expanding applications to wound dressings and tissue engineering [80]. However, natural gels degrade 20–30% faster in humid environments, and thus require hybrid formulations for longevity [81]. These superior mechanical properties—including high tensile strength (up to 2.25 MPa), exceptional stretchability, and toughness—directly translate into enhanced probe stability, resistance to deformation during scanning, and prolonged structural integrity. Consequently, such gel pads reduce air-bubble formation and dehydration artifacts, leading to improved near-field image resolution and greater reliability in prolonged diagnostic and intraoperative ultrasound procedures.

Chitosan-based hydrogel pads are gaining attention in ultrasound imaging due to their transparency, porosity, strong adhesion, and biocompatibility. Chen et al. [82] optimized a chitosan-based hydrogel using chitosan, 2-acrylamido-2-methylpropanesulfonic acid, and N-isopropylacrylamide, achieving a transparent, porous pad with high tensile strength and good swelling properties. This pad provided ultrasound images statistically indistinguishable from standard coupling agents, maintained performance for at least 10 days, and was non-irritating for repeated clinical use. Li et al. [83] developed a PAA-NHS/catechol-functionalized chitosan hydrogel with robust wet adhesion and high mechanical strength (tensile strength ~630 kPa, fracture strain ~1950%), which is promising for stable coupling in damp environments like ultrasound procedures. Zhang et al. [84] created a quaternized chitosan/poly(ionic liquid) hydrogel with excellent adhesion, plasticity, and recyclability, maintaining over 70% adhesion to skin after 10 cycles, which supports repeated ultrasound imaging sessions. Deng et al. [85] introduced a methacrylate chitosan/hyaluronic acid double-network hydrogel, which forms a transparent, cohesive pad with strong wet-tissue adhesion (bursting pressure up to 623 mmHg), suitable for stable probe contact during imaging. Li et al. [86] reported a polyacrylamide–chitosan–Al^3+^ double-network hydrogel with high transparency (>90%), strong self-adhesion, and excellent mechanical properties (strain up to 1040%, toughness 730 kJ/m^3^), making it ideal for flexible, skin-conforming ultrasound pads. Cui et al. [87] fabricated a chitosan/poly(acrylic acid) double-network nanocomposite hydrogel with tunable mechanical properties (fracture stress up to 1.2 MPa, strain up to 800%) and strong adhesion to skin and other substrates, supporting stable and conformable ultrasound coupling. Jiang et al. [88] developed a chitosan/poly(acrylamide-acrylic acid) hydrogel crosslinked with Al^3+^, achieving tensile strength of 0.54 MPa and elongation at break of 2203.7%, ensuring durability and flexibility for imaging applications. The combination of high transparency, strong wet adhesion, and robust mechanical performance in these chitosan- or nanocomposite-reinforced hydrogels enables stable coupling on high-curvature or moist anatomical sites (e.g., intraoral, neck, or musculoskeletal regions). This results in better acoustic transmission, reduced operator fatigue, and higher patient comfort compared to traditional liquid gels.

Bilayer hydrogel pads are a type of pad that has a rigid layer and a compliant or conformal layer to contact with the skin; therefore, they are particularly useful in ultrasound imaging over high-curvature regions like elbows, fingers, and skin tumors. This design enables the pad to be externally compression bearing, spread stress, and move the probes smoothly without damaging the skin. The bilayer hydrogel pads were found in clinical work to support higher-quality imaging of thyroid nodules and skin tumors than liquid and single-layer solid gels, and were shown to conform better and image better on curved surfaces [89]. Peek et al. [90] prepared a bilayer phantom of polyvinyl alcohol hydrogel and ballistic gel to simulate the layered human tissue of the high-intensity focused ultrasound (HIFU). The bilayer phantom simulated the errors and deformities of actual tissue and offered a testing background to study the distortion of the ultrasound fields and create corrective algorithms for therapeutic and diagnostic ultrasound. Pan et al. [91] presented a soft armor-based hydrogel having an outer layer that was hydrophobic with a hydrogel matrix that ensured strong adhesion to wet tissue and high-contrast ultrasound imaging in intraoral practice, which showed a potential for stable anchoring and imaging in very moist and anatomically complex locations. By integrating a rigid load-bearing layer with a soft conformal layer, bilayer hydrogel pads effectively distribute pressure and maintain intimate contact with irregular surfaces. This structure–property relationship significantly improves image quality for superficial structures and skin tumors while minimizing pressure-related artifacts and enhancing patient safety during extended examinations.

The gelatin and soft solid gel pad are increasingly used in ultrasound imaging as a coupling medium to avoid the drying up and re-application problems that accompany common gels. A solid gel pad was pioneered by Kim et al. [92] as an intraoperative ultrasound medium for reducing the facial bones following fracture. It was light, soft, and simple to operate, giving high-quality images to enhance and be able to guarantee better performance in closed reduction surgery due to the increased contact of the probe to the skin surface, and provided images of curved surfaces on the face. According to Tsui et al. [93], a flexible gel pad, which was melted wax, offered great images and great stability of the probe position even when scanning other irregular surfaces such as the anterior neck. The advantage of the gel pad was the greater scanning field and uniform images compared to a regular liquid gel. It may be utilized in clinical/educational ultrasound settings. Alarcón et al. [94] utilized the gelatin samples to measure the surface wave propagation through ultrafast ultrasound imaging. They could show how gelatin was suitable for advanced imaging research and elastography. In another study, Rossello et al. [95] applied gelatin in high-speed ultrasound imaging to study bubbly flows and shear waves, which again increases the use of soft solid gels in experimental and diagnostic ultrasound.

Wax and paraffin gel pads are low-cost, accessible alternatives to standard ultrasound gels, particularly useful in musculoskeletal and educational imaging. Phani et al. [96] developed and validated gel-wax phantoms for diagnostic ultrasound, reporting acoustic properties close to soft tissue: speed of sound (crus) 1431.4 m/s, mass density 0.87 g/cm^3^, acoustic impedance 1.24 MRayl, and attenuation coefficient 0.7–0.98 dB/cm/MHz at 22 °C. The phantoms showed a maximum measurement error of 7.1% and only 1.8% volume loss over 62 weeks, indicating high stability and suitability for repeated use in scanner evaluation and training [97]. Vieira et al. [98] investigated paraffin-gel waxes as tissue-mimicking materials for ultrasound-guided breast biopsy phantoms, achieving a speed of sound between 1425.4 and 1480.3 m/s, attenuation coefficients from 0.32 to 2.04 dB/cm at 7.5 MHz, and Young’s modulus between 14.7 and 34.9 kPa. These phantoms did not dehydrate, provided adequate needle penetration, and maintained geometry over time, making them effective for procedural training. Maneas et al. [99] used gel wax with paraffin and glass spheres to create anatomically realistic ultrasound phantoms, tuning attenuation from 0.72 to 2.91 dB/cm at 3 MHz and 6.84 to 26.63 dB/cm at 10 MHz, with Young’s modulus of 17.4 ± 1.4 kPa.

Medical polymer and commercial reusable gel pads are increasingly used in specialized and resource-limited ultrasound applications, offering both safety and cost-effectiveness. 3M™ Defib-Pads serve as a reusable alternative, providing interpretable images in cardiac, abdominal, and nerve ultrasound under various environmental conditions, except after prolonged air exposure, which is reversible with rinsing [25]. In pediatric extracorporeal shock wave lithotripsy (ESWL), reusable gel pads like 3M™ Defib-Pads have been utilized to enhance the transmission of shock waves, ensuring optimal treatment efficacy. These gel pads maintain consistent performance, providing stable contact during the procedure and reducing discomfort, while also being a cost-effective and environmentally friendly alternative in resource-limited settings [100]. In pediatric extracorporeal shock wave lithotripsy (ESWL), He et al. [38] used a 4 × 5 × 10 cm medical polymer gel pad in 21 infants, achieving an 85.7% lithotripsy success rate with no probe abnormalities or post-procedure complications. The average stone size was 0.60 ± 0.21 cm, and the average surgical time was 39.8 ± 13.8 min, demonstrating the pad’s efficacy in protecting the probe from shock wave energy while maintaining imaging accuracy. Jain [37] designed a polymer transducing pad using divinyl, acrylic acid, acrylate monomers, and poly(ethylene glycol), which produced ultrasound images similar to commercial gel and offered prolonged reusability, making it suitable for low-resource settings. Vengerova et al. [101] reported on medical polymer gels like REPAC and DIAGEL, which have very low sound damping (about 0.001 dB/cm) and can maintain 100% contact for up to 20 min, outperforming other gels in clinical practice. By comparing polymer hydro gels, emulsions, and oil gels, it was found that polymer hydro gels had the highest moisture evaporation. Still, it provided good skin lubricity and was suitable for diagnostic ultrasound transmission [102]. These studies highlight that medical polymer and commercial reusable gel pads can deliver reliable image quality, safety, and reusability, making them valuable for both routine and specialized ultrasound imaging, especially where standard gels are impractical or unavailable.

Standoff pads and improvised gel pads, such as water baths and water-filled patient belongings bags (PBBs), are widely used to improve ultrasound imaging of superficial soft tissue structures and foreign bodies, especially in emergency and resource-limited settings. In a 2025 prospective study involving 18 emergency physicians and 288 scans, PBBs and water baths demonstrated the highest accuracy for foreign body detection, followed by gel and saline bag standoffs. Gel was significantly more accurate than saline bags (*p* = 0.0120), and PBBs were found to be a viable, practical alternative for soft tissue imaging when standard materials are unavailable [103]. Corvino et al. [104] have shown that gel standoff pads can significantly enhance the detection of peri- or intra-lesional blood flow in superficial skin lesions using Doppler imaging, increasing detection rates from 56% without a pad to 84% with a pad (*p* < 0.001). Liquid and gel standoff pads also affect image quality metrics such as lateral resolution and near-field intensity, with differences primarily due to probe height above the skin rather than the pad material itself [105]. Improvised standoff devices, such as intravenous fluid bags of various sizes, have been shown to provide image quality comparable to commercial standoff pads for musculoskeletal ultrasound, particularly for soft tissue foreign body models, with larger bags sometimes outperforming standard devices [106]. These findings support the use of both commercial and improvised standoff solutions to optimize ultrasound imaging in diverse clinical scenarios.

Double-network and nanocomposite hydrogels exhibit excellent mechanical and acoustic properties in laboratory settings (see Section 1 for coupling principles); however, their long-term acoustic stability after repeated sterilization and their performance in humid clinical environments remain largely unvalidated.

While polyacrylamide (PAM) hydrogels consistently demonstrate superior tensile strength (up to 1.98 MPa) and water stability compared with natural hydrogels (e.g., chitosan or gelatin, which degrade 20–30% faster), they exhibit higher production costs and limited scalability [20,44]. Silicone elastomers offer excellent long-term chemical stability but lack the self-healing and tunable elasticity of DN hydrogels, resulting in poorer performance on high-curvature anatomy. Nanocomposite reinforcements improve mechanical toughness across all classes but introduce concerns regarding nanoparticle leaching and regulatory hurdles. These discrepancies highlight that no single material currently satisfies all clinical requirements (acoustic performance, biocompatibility, cost, and sustainability) simultaneously, underscoring the need for hybrid formulations.

Table 2 is a heterogeneous and dynamic category of coupling media in medical diagnostics, where each of the variants is aimed at meeting certain clinical demands and technological requirements. In one instance, aptamer cross-linked hydrogel pads have allowed point-of-care testing of several types of analytes, such as drugs in urine, with a quantitative readout of a visual, sensitive and specific, distance-based format, [107]. Aqueous flexible gel pads called stand-off gel pads have been reported to be highly effective in detecting peri- or intra-lesional blood flow in the skin lesions of superficial skin lesions during the process of Doppler ultrasound, and hence improve the diagnostic accuracy of skin pathologies [104]. Gel pads in breast imaging do not affect the repeatability or the diagnostic utility of shear wave elastography, and gel pads are particularly beneficial to enhance the near-field resolution and quality of the image of superficial lesions [71].

Nanocomposite hydrogel pads that include nanoparticles provide multifunctional diagnostics and therapeutic platforms that respond to stimuli (e.g., ultrasound) to allow controlled release of drugs, tissue engineering, and real-time biosensing [108]. Ionic liquid gel pads are embedded in textile bioelectrode arrays to offer low-impedance, high-adhesion, and reliable whole-body electromyography monitoring in the field of wearable technology. Polymer gel pads used in medicine have also been applied to pediatric extracorporeal shock wave lithotripsy, which insulates the ultrasound probe from the shock wave and does not affect the quality of imaging [38].

**Figure 4:**
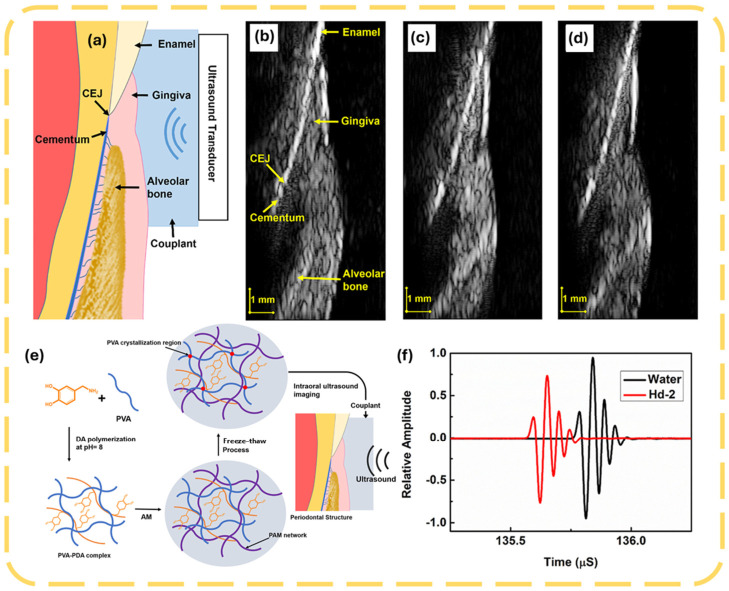
Intraoral ultrasound imaging using a bilayer hydrogel couplant. (**a**) Schematic of the periodontal structure and ultrasound setup with the hydrogel couplant. (**b**–**d**) Representative ultrasound images showing clear visualization of enamel, gingiva, cemento-enamel junction (CEJ), cementum, and alveolar bone. (**e**) Synthesis and molecular structure of the PVA-PDA hydrogel (Hd-2) via dopamine polymerization and freeze-thaw cycles. (**f**) Comparison of ultrasound echo signals between water and the Hd-2 hydrogel couplant. Reprinted from ACS with permission [113].

Other specialized gel pads are those used in automated breast sonography, to decrease patient pain and increase scan area without image degradation [109]; there are also thin gel pads that optimally transmit ultrasound in therapeutic use over tendons and superficial regions [110]. The wearable pads based on metamaterials increase the electromagnetic coupling of implantable and wearable diagnostic devices, which increases the signal penetration and communication with biological tissues [111]. Also, pressure-distributing gel pads have been made large, using freehand 3D ultrasonography, to minimize the effects of probe pressure on artifact and enhance the sensitivity of measuring the volume of muscles [112]. These inventions show that gel pads are part and parcel of enhancing diagnostic imaging, wearable monitoring, and therapeutic procedures, and the current research aims at enhancing their functionality, patient comfort, and versatility to diverse clinical conditions. Figure 4 illustrates the potential of polyacrylamide/alginate double-network (DN) hydrogels as advanced ultrasound gel pads. Yi et al. [113] demonstrated that these hydrogels provide excellent acoustic coupling for intraoral imaging. Figure 4a,b show high-quality ultrasound images of porcine mandibular incisors using the DN hydrogel, with clear visualization of key structures including enamel, gingiva, cementum, and alveolar bone. Figure 4c,d compare the hydrogel’s performance with commercial Aquaflex and water, respectively, revealing comparable image quality while offering superior mechanical stability and lower cytotoxicity. The synthesis route of the hydrogel via freeze–thaw cycling and dopamine modification is presented in Figure 4e, while Figure 4f quantifies its acoustic performance, showing more consistent signal amplitude compared to water. The DN hydrogel combines acoustic performance comparable to commercial pads with significantly better mechanical strength, water stability, and biocompatibility, making it particularly suitable for intraoral and high-curvature ultrasound applications.

**Table 2:** Types of gel pads and their applications in medical diagnostics.

Type of Gel Pad	Material	Primary Application(s)	Benefits	References
**Automated Breast Sonography Gel Pad**	Medical-grade silicone elastomer (polydimethylsiloxane, PDMS) hydrogel	Automated breast ultrasound	Reduces pain, expands scan coverage, maintains image quality	[99]
**Thin Gel Pad**	Medical-grade polydimethylsiloxane (PDMS) or polyurethane (PU) hydrogel, thickness <1 cm, highly flexible	Therapeutic ultrasound over tendons, superficial areas	Transmits ultrasound efficiently, suitable for hands/ankles	[101]
**Metamaterial-Inspired Wearable Pad**	Polydimethylsiloxane (PDMS) dielectric matrix embedded with metallic loops	Enhancing EM coupling for implantable/wearable devices	Improves signal penetration, flexible, robust for diagnostics/communication	[102]
**Freehand 3D Ultrasound Gel Pad**	Large-volume medical-grade polyvinyl alcohol (PVA) or agarose hydrogel	Muscle volume measurement (e.g., gastrocnemius)	Reduces probe pressure artifacts, improves volumetric accuracy	[103]
**Stand-off Gel Pad**	Aqueous polyacrylamide (PAM) hydrogel, flexible and disposable	Doppler ultrasound of superficial skin lesions	Enhances detection of peri- or intra-lesional blood flow, improves diagnostic accuracy	[71,104]
**Hydrogel Pad (Sweet Hydrogel)**	Aptamer cross-linked polyacrylamide hydrogel	Point-of-care testing, quantitative diagnostics (e.g., cocaine in urine)	Target-responsive, disposable, visual quantitative readout, low cost	[107]
**Nanocomposite Hydrogel Pad**	Stimuli-responsive chitosan or polyacrylamide hydrogel reinforced with silica/gold nanoparticles	Drug delivery, tissue engineering, biosensing, ultrasound-triggered diagnostics	Responds to stimuli (ultrasound, pH, etc.), enables real-time imaging and controlled release	[108]
**Polymer Gel Pad**	Medical-grade thermoplastic polyurethane (TPU) or silicone polymer	Ultrasound monitoring during extracorporeal shock wave lithotripsy (ESWL) in infants	Protects probe from shock waves, maintains imaging quality, increases probe–skin distance	[114]
**GelMA Hydrogel Pad**	Gelatin methacryloyl (GelMA) hydrogel	Wearable biosensors, physiological monitoring	High sensitivity, flexibility, long-term stability, suitable for motion/pressure sensing	[115]
**Ionic Liquid Gel Pad**	Ionic liquid (e.g., 1-ethyl-3-methylimidazolium tetrafluoroborate) gel embedded in textile matrix	Wearable bioelectrode arrays for electromyography (EMG)	Low impedance, strong adhesion, suitable for whole-body monitoring	[116]
**Laryngeal Ultrasound Gel Pad**	Soft conformable medical-grade carbomer or silicone-based hydrogel	Vocal cord evaluation in neck surgery	Improves visualization, especially in calcified cartilage, increases diagnostic efficacy	[117]

## 5. Materials Science and Functional Enhancement in Gel Pad Development

The performance and versatility of ultrasound gel pads are fundamentally determined by the materials from which they are engineered. Advances in materials science have enabled the design of coupling interfaces that balance acoustic efficiency, mechanical stability, patient comfort, and environmental responsibility. This section examines synthetic and natural polymers, hydrogels, silicones, and nanotechnology-enhanced systems, with emphasis on polymer chemistry, crosslinking mechanisms, network architecture, structure–property relationships, and smart functionalization. These advances contribute to the further growth of gel pads in modern medical imaging practices.

### 5.1. Polymeric and Natural Hydrogel Systems

The development of ultrasound gel pads is closely connected with advances in polymer chemistry. Single-network hydrogels rely primarily on covalent or ionic crosslinking of a single polymer chain, resulting in relatively brittle structures with limited energy dissipation. In contrast, double-network (DN) hydrogels combine a brittle first network (e.g., polyacrylamide) with a ductile second network (e.g., alginate or chitosan), enabling efficient energy dissipation through sacrificial bond breaking and achieving superior toughness and stretchability. This architecture directly improves structure–property relationships: higher tensile strength, elasticity, and acoustic impedance while maintaining low attenuation. Combining polyacrylamide and alginate in a double-network (DN) hydrogel system, synthesized rapidly by ultrasound cavitation, presents hitherto unexplored properties of using ultrasound cavitation to produce hydrogel systems with exceptional fracture-toughness, optical transparency, and anti-freeze capability, making it both clinically and extreme-environment friendly. Poly(ethylene glycol) (PEG)-based hierarchical gels with micelle-crosslinked structures provide robust adhesion, self-healing ability, and tunable mechanics, enabling reusable and durable ultrasound pads [118,119].

Similar ultrasound-responsive synthetic polymers are being developed as smart, non-invasive drug-delivery vehicles for tumor therapy and transdermal platforms [120]. Li et al. [121] discovered tunneling developments in frontal polymerization (FP) with ultrasound to prepare nanocomposites, gradient materials, and interpenetrating polymer networks. FP allows fabricating functional gels with fast, energy-efficient production and specific properties, thus widening the design space of ultrasound gel pads. Equally, piezo-polymerization that makes use of piezoelectric nanoparticles to propel disulfide-crosslinked adaptable networks presents recyclable and mechanically sensitive gel systems [122]. Hybrid approaches based on the use of POSS-based gels increase strength, thermal resiliency, and microstructural tunability, extending properties to imaging and therapy. In addition, accuracy made possible by controlled radical polymerization enables the incorporation of functional groups and custom architectures, creating flexible hybrid gels to suit next-generation ultrasound applications [123]. Viscoelasticity, swelling, and the efficiency of acoustic transmission directly depend on structural innovations in block copolymers, branched networks, and densities of cross-linkages [124].

The use of gels based on natural polymers is of particular interest as a substitute for traditional synthetic formulations, as they are biodegradable and cost-effective. Wu et al. [125] revealed that poly(vinyl alcohol) (PVA) hydrogels can be controlled through the Hofmeister effect to tune mechanical performance between tensile strengths of 50 ± 9 kPa to 15 ± 1 MPa, elongations of 300% to 2100%, and moduli of 24 ± 2 to 2500 ± 140 kPa with high biocompatibility. This tunability is of particular concern to ultrasound coupling gels, where mechanical compliance and acoustic impedance must be precisely matched to skin–probe interactions.

While DN hydrogels achieve excellent mechanical and acoustic properties in laboratory settings, their long-term acoustic stability after repeated sterilization and performance in humid clinical environments remains largely unvalidated. Natural-polymer variants degrade 20–30% faster than synthetic counterparts, representing a key trade-off between biocompatibility and durability that warrants further hybrid formulation research.

**Figure 5:**
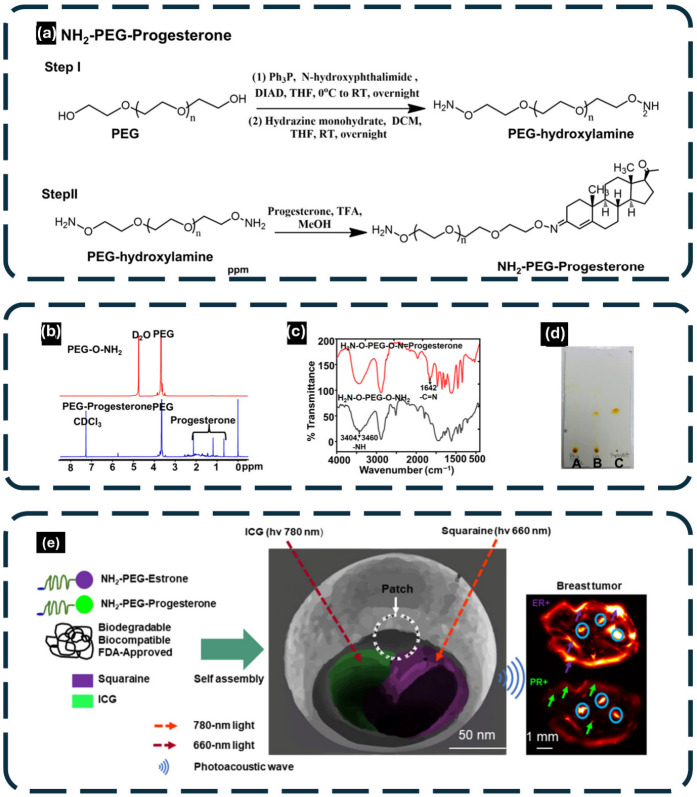
Synthesis and characterization of NH_2_-PEG-Progesterone for a biodegradable photoacoustic imaging patch. (**a**) Two-step synthetic route for NH_2_-PEG-Progesterone. (**b**) ^1^H NMR spectrum confirming successful conjugation. (**c**) FTIR spectrum of the conjugate. (**d**) Photograph of the self-assembled formulations. (**e**) Schematic illustration of the self-assembly of NH_2_-PEG-Estrone/Progesterone with squaraine and ICG into a biocompatible patch for dual-wavelength (660 nm and 780 nm) photoacoustic imaging of ER^+^/PR^+^ breast tumors, enabling enhanced molecular-specific intratumor visualization. Reprinted from ACS with permission [126].

Li et al. [126] brought a non-invasive hybrid imaging platform that combines multitargeted polymer-based contrast agents with photoacoustic computed tomography to detect intratumor heterogeneity in breast cancer, as shown in Figure 5. The use of specific polymers in this figure as the ultrasound gel pads should allow improved imaging to be performed. Figure 5a shows how NH_2_-PEG-Progesterone, which is one of the components, is synthesized, whereas Figure 5b,c give NMR and FTIR confirmation of its molecular structure to make it compatible with ultrasound transducers. Figure 5d,e depict how the biodegradable NH_2_-PEG-Estrone and NH_2_-PEG-Progesterone self-assemble with squaraine and ICG into photoacoustic emissions at 660 nm and 780 nm light respectively. These phenomena increase molecular specificity of the estrogen and progesterone receptor-positive tumors, and thus the clinical outcome is improved. The time-lapse fluorescence and photoacoustic imaging of ER+ and PR+ breast cancer cells confirm the real-time diagnostic ability of the system and the use of the specific polymer properties to enhance the acoustic coupling and the imaging resolution to identify the intratumor heterogeneity in a more accurate manner.

### 5.2. Nanotechnology and Smart Functionalization

Over the last several years, nanotechnology’s introduction in the development of ultrasound gel pads has resulted in major improvements in performance, functionality, and clinical potential. Nanoparticles possess distinct physical and chemical properties that can be exploited to overcome the drawbacks of conventional gel pads.

Nanocomposite reinforcement occurs primarily through hydrogen bonding and the physical adsorption of nanoparticles (e.g., graphene oxide, silver, zinc oxide) onto the polymer matrix. This mechanism increases tensile strength and toughness while enabling acoustic impedance tuning (closer matching to soft tissue) and ultrasound-triggered responsiveness (cavitation-induced drug release or shape change). For example, zinc oxide (ZnO) nanoparticles embedded in hydrogel matrices produce extremely elastic nanocomposites with stretchability up to 260%, excellent biocompatibility, and no acute toxicity in cell studies, serving as efficient ultrasound contrast agents and wireless strain sensors [127]. Silica nanoparticles, together with supplementary polymer reinforcement, improve the integrity of the polymer matrix, thereby enhancing solidification and effectively preventing delamination during prolonged imaging sessions [128].

Selenium nanoparticles implanted into composite hydrogels demonstrate potent antioxidant and anti-inflammatory action that can be controllably regulated by ultrasound, accelerating healing in bone defect models [129]. Silver nanoparticles impart natural antimicrobial effects, reducing cross-infection risk in clinical settings [130,131,132,133].

**Figure 6:**
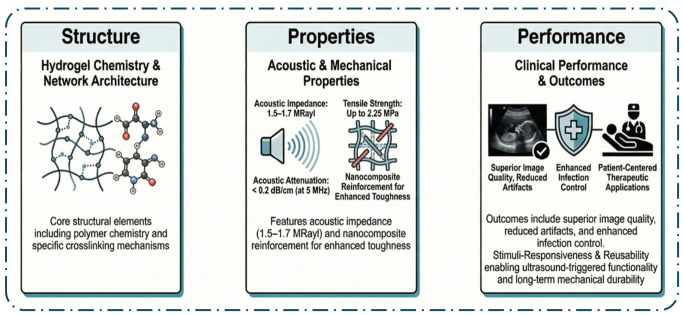
Structure–property–performance of advanced hydrogel gel pads. (**Left**) Hydrogel chemistry and network architecture. (**Center**) Acoustic and mechanical properties (impedance 1.5–1.7 MRayl, tensile strength up to 2.25 MPa, low attenuation). (**Right**) Clinical performance showing superior image quality, reduced artifacts, and enhanced patient outcomes.

Although nanocomposite hydrogels show promising reinforcement, acoustic modulation, and stimuli-responsiveness in vitro, most data are derived from small-scale or phantom studies. Clinical translation is limited by long-term nanoparticle leaching, regulatory requirements for biocompatibility, and scalability of uniform dispersion, highlighting the need for larger in vivo validation. The integrated relationships between material structure, resulting properties, and ultimate clinical performance are summarized in Figure 6.

**Figure 7:**
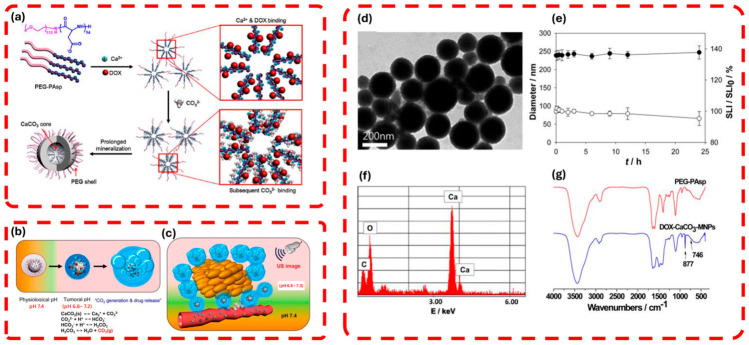
Synthesis, pH-responsive behavior, and characterization of doxorubicin-loaded calcium carbonate mineralized nanoparticles (DOX-CaCO_3_-MNPs). (**a**) Schematic of PEG-PAsp assembly, Ca^2+^/DOX binding, and subsequent mineralization. (**b**) pH-triggered CO_2_ generation and drug release mechanism. (**c**) Ultrasound imaging illustration at tumoral pH. (**d**) TEM image of nanoparticles. (**e**) Colloidal stability over 24 h. (**f**) EDS spectrum confirming Ca content. (**g**) FTIR spectra of PEG-PAsp and DOX-CaCO_3_-MNPs. Reprinted from ACS with permission [134].

Min et al. [134] pioneer a theranostic approach using pH-controlled gas-generating mineralized nanoparticles for ultrasound imaging and cancer therapy, as shown in Figure 7, where the integration of nanotechnology into gel pads enhances imaging capabilities. Figure 7a outlines the synthesis of DOX-CaCO_3_-MNPs via PEG-PAsp templated mineralization, creating a stable nanoparticle core that improves acoustic coupling and durability, akin to nanoparticle-enhanced gel pads. Figure 7b illustrates the CO_2_ generation and DOX release under tumoral acidic conditions, mirroring how nanotechnology enables pH-responsive gel pads to optimize acoustic transmission. Figure 7c demonstrates enhanced ultrasound echogenicity in tumors, highlighting the potential for nanoparticle-laden gels to improve imaging resolution. Figure 7d,e provide TEM and EDS data confirming nanoparticle structure and calcium content, supporting the use of such materials in gel pads for tailored mechanical properties. Figure 7f,g show size stability and FTIR spectra, underscoring the precision of nanofabrication to develop smart gel pads with therapeutic and imaging functionalities.

### 5.3. Biocompatibility and Sustainability

The biocompatibility of ultrasound gel pads is also important since the materials come into direct contact with the skin of the patients during imaging processes. Biocompatibility will imply that the gel pad materials should not be irritating, allergic, or toxic. Recent studies have also placed an emphasis on the application of synthetic and natural polyacrylamide, alginate, chitosan and hydrogel. One of the most popular among them is known to be safe and can be used with biological tissues. Indicatively, the study by Buxaderas et al. [135] showed that optimized ultrasound-based hydrogel-based phenylalanine derivatives had high cell viability rates of more than 95 percent, which proves their suitability in biomedical applications. In the same light, polyacrylamide/alginate double-network hydrogels have been found to be less cytotoxic than commercial gel pads, thus becoming favorable candidates to intraoral ultrasound imaging. Natural and bio-sourced polymers are used more actively because of their biodegradable nature and less harmful effect on the environment. Chitosan is one biopolymer that can be mentioned due to its sustainability, flexibility, and biocompatibility, which makes it appropriate in wearable and disposable ultrasound devices [136]. Biopolymeric gels offer advantages such as renewable raw materials, energy-efficient fabrication, and superior biodegradability, positioning them as key materials for sustainable medical applications [137]. These materials not only meet safety standards but also align with the growing demand for environmentally responsible healthcare products.

Sustainability is a growing priority in gel pad development. Biodegradable polymers such as chitosan, alginate, and gelatin derived from food-waste sources offer lower lifecycle impact through reduced plastic waste and renewable sourcing. For example, tamarind-seed-gum pads demonstrate comparable acoustic performance to synthetic gels while being fully biodegradable within 6–12 months in soil [19]. Preliminary lifecycle analyses indicate up to 65% lower carbon footprint compared with conventional polyacrylamide gels when locally sourced raw materials are used. However, comprehensive cradle-to-grave assessments remain limited. Salmon et al. [9] applied human-centered design to develop user-friendly ultrasound gel based on cassava root flour and bula at low costs but without damaging the image quality, which is a viable solution in resource-deprived environments. Gel pads have been enhanced by materials science inventions to enhance their performance and sustainability.

Fabrication is a challenging task, and ultrasound-assisted methods enable the manipulation of biopolymer structures in a precise manner, with a positive effect on mechanical and acoustic characteristics, without altering biocompatibility. The study by Cai et al. [138] examined the potential of ultrasonic cavitation to modify natural polymers and produce materials with better strength, flexibility, and environmental friendliness, which can find use in the medical world. These innovations assist in the creation of high-performing and sustainable gel pads.

The use of the other natural gels like Aloe vera as an ultrasound coupling agent has also been studied comparatively; Aloe vera gel not only provides acoustic transmission comparable to or better than commercial gels, but also demonstrates high biocompatibility and safety with biological tissues [139]. This underscores the possibility of using plant-based gels as effective, sustainable and safe alternatives within clinical practice. Another sustainable alternative to traditional transducing pads that are design-based is the use of polymer-based transducing pads, especially in low-income or resource-deprived environments. Such innovations are crucial for expanding access to ultrasound diagnostics while minimizing environmental impact [140,141,142].

Taken together, the literature reveals clear trade-offs: synthetic DN hydrogels excel in mechanical toughness and acoustic transmission but are less sustainable and more expensive, whereas natural-polymer hydrogels offer superior biodegradability and biocompatibility at the expense of lower long-term stability. Silicone-based systems remain the clinical standard for reusability but fall short in self-healing and adaptability to irregular surfaces. These inconsistencies highlight a critical gap—the absence of a single material that optimally balances acoustic performance, patient safety, environmental impact, and cost-effectiveness.

Polyacrylamide, Hydrogel and Silicone are some of the materials that are discussed in Table 3 since each material shows its own strengths and weaknesses when it comes to biomedical and industrial applications of gel pads. Silicone gel pads are not as mechanically strong, water stable, or biocompatible as polyacrylamide-based gel pads, particularly those that have a double-network hydrogel structure. Indicatively, polyacrylamide/alginate double-network hydrogels have enhanced stability in water, mechanical properties and reduced cytotoxicity that makes them very appropriate in intraoral ultrasound imaging [18]. Likewise, nanofibril-reinforced iron ion-reinforced hydrogel of polyacrylic acid has high tensile strength (up to 1.98 MPa), high fracture elongation (up to 838.8), and stable swelling at different pH and saline regimes and, additionally, has antibacterial and antioxidant properties in use as an absorbent pad [143]. Hydrogels, especially those of a double-network or interpenetrating network structure, are very stretchable, tough and may be programmable to enhance self-healing rapidly and strong self-bonding. They are attained by mixtures of irreversible and reversible cross-linking, and host-guest reactions, which enable the hydrogels to retain their shape and functionality following repeated mechanical stress [144,145]. There is an example of alginate-based cyclodextrin/Azo-polyacrylamide Hydrogels whose self-healing behavior is exhibited in the presence of light and the ability to regain high elongation after healing, promising the implementation of load bearing and support atoms. By contrast, silicone gel pads, though commonly used in commercial applications due to their chemical and thermal stability and inertness, typically do not have tunable mechanical characteristics, self-healing, or high adhesion characteristics of the more complex hydrogel and polyacryl amide systems.

## 6. Gel Pad Manufacturing and Production Techniques

The production of ultrasound gel pads has evolved into a sophisticated field that combines traditional methods with advanced technologies. Manufacturing processes directly determine structural integrity, acoustic performance, patient comfort, and scalability. From conventional casting and injection molding to emerging additive manufacturing techniques such as 3D printing and continuous liquid interface production (CLIP), each approach offers distinct advantages and limitations. This section examines these techniques with emphasis on quality control, regulatory compliance, and the ability to produce customized, multifunctional gel pads for clinical and therapeutic applications.

### 6.1. Conventional Manufacturing Approaches

Casting is a simple and cost-effective method suitable for small-scale or custom production. However, traditional casting is time-consuming and often results in inconsistent thickness and material properties, which can compromise acoustic uniformity and mechanical performance [153,154,155]. Continuous casting has largely overcome these limitations by enabling higher throughput and improved uniformity. For example, Leite et al. [156] demonstrated that continuous casting increased productivity by at least 1000-fold compared with bench-scale casting while enhancing the mechanical and barrier properties of gelatin-based bio-nanocomposites.

Injection molding offers significant advantages for large-scale production. Molten polymer or gel is injected under pressure into a mold, providing excellent control over shape, thickness, and surface finish with high repeatability and low labor requirements. This makes injection molding particularly suitable for high-volume manufacturing of pads requiring strict tolerances [157,158,159,160]. Peixoto et al. [160] highlighted that injection molding is the most efficient technology for producing complex, high-precision polymer components, provided processing parameters and mold quality are carefully controlled. Although conventional methods are cost-effective for mass production, they lack flexibility for patient-specific customization and require expensive custom molds for new designs. This limits their suitability for low-volume or highly differentiated clinical applications.

### 6.2. Additive Manufacturing and Emerging Fabrication Technologies

Additive manufacturing, commonly known as 3D printing, represents a major advancement by enabling the layer-by-layer deposition of materials to create complex, patient-specific geometries. This technology is particularly valuable for producing gel pads tailored to individual anatomy or functional requirements in medical and wearable devices. Chen et al. [161] showed that carbomer-modified hydrogel inks can be 3D-printed into multifunctional hydrogels with excellent mechanical properties and biocompatibility, opening new possibilities for integrated soft devices. Similarly, Li et al. [162] developed a digital light processing method to print tannic acid-based gels with enhanced toughness, anti-dehydration, and antibacterial properties suitable for tissue engineering and wearable applications.

The 3D printing customization opportunity finds additional emphasis in the food industry, where it is possible to create gels with nutritional and textural characteristics. García-Segovia et al. [163] and Riantiningtyas et al. [164] further demonstrated the potential of 3D printing for customizing gel structures, including high-protein yogurt-based gels optimized for physical and sensory properties. Cui et al. [165] emphasized the importance of shear-thinning and stable flow properties in food-grade polymer inks for successful extrusion-based printing.

While 3D printing enables unparalleled customization and functional integration, it remains slower and more expensive than traditional methods, limiting scalability for high-volume production. Material consistency and long-term mechanical stability of printed gels also require further validation before widespread clinical adoption.

### 6.3. Functional Customization and Smart Material Integration

Modern manufacturing allows the creation of gel pads with patient-specific and functionally enhanced designs. Standard gel pads are universal, but anatomical variations (high curvature, irregular surfaces) often reduce coupling efficiency. Bilayer hydrogel pads with a rigid outer layer and compliant inner layer conform better to complex surfaces such as elbows, fingers, and skin tumors, providing superior acoustic coupling and reduced pressure artifacts [89]. Likewise, with respect to abdominal aortic aneurysm, the application of standardized gel pads has also made transducer pressure and tissue deformation measurements more reliable, which contributes to the necessity to employ patient-specific solutions to reduce measurement error and enhance the reliability of the diagnostic results [166].

Using gel pads that were designed for specific anatomical areas, like the abdomen, custom gel pads also incorporate smart functionalities such as thermal control, antimicrobial properties, and shape-memory effects. Phase-change materials or thermally conductive fillers enable temperature regulation for physiotherapy applications, while silver nanoparticles provide built-in infection control [167,168]. Addition of functional agents or creation of microstructures in the gel matrix also further expands the list of applications that can be sought with such materials and include not only tissue engineering applications but also advanced diagnostics and smart-gel systems [167,169]. Huang et al. [169] produced alginate/polyacrylamide hydrogels via continuous liquid interface production with reversible shape-memory properties, allowing pads to adapt to anatomical needs and reform after use. Kim et al. [17] developed a soft solid gelatin gel that overcomes the drying and impedance mismatch issues of traditional gels, showing superior imaging performance. Functional customization greatly improves patient comfort and diagnostic accuracy, yet most studies remain proof-of-concept with small sample sizes. Long-term durability, sterilization compatibility, and cost-effectiveness of smart features need further clinical validation.

### 6.4. Scalability and Quality Control

Despite technological advances, scaling the production of customized gel pads presents significant challenges. Material consistency is critical: variations in polymer composition, water content, or additive distribution can alter acoustic impedance, mechanical properties, and imaging performance. Strict quality control protocols, including quantitative assessment of pad flatness and uniformity, are essential [170,171]. Vo et al. [171] showed that a 3D-printed agarose micropad system significantly improved consistency and flatness, underscoring the importance of rigorous quality management as production volumes increase.

Cost remains a major barrier. Injection molding requires expensive custom molds, while advanced materials and functional additives (e.g., antimicrobial agents) increase raw-material expenses. 3D printing, although highly flexible, is currently less efficient and more costly for large-scale production than conventional methods [168,172,173]. Supply-chain stability for specialized polymers and biocompatible additives further complicates manufacturing [171,174,175].

Advances in 3D printing and continuous casting enable patient-specific customization, yet scalability and regulatory compliance remain significant barriers. Most fabrication studies are proof-of-concept with small sample sizes, limiting confidence in industrial translation [176]. Future work should focus on hybrid manufacturing approaches that combine the precision of additive methods with the throughput of conventional techniques to achieve both customization and cost-effectiveness.

**Figure 8:**
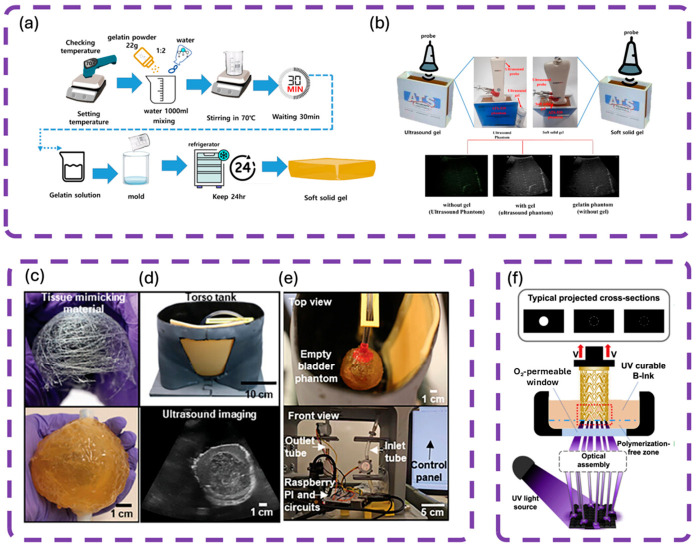
Fabrication and characterization of soft solid gelatin gel pads for ultrasound imaging. (**a**) Schematic of the preparation process of the soft solid gelatin gel. (**b**) Experimental setup comparing commercial ultrasound gel and soft solid gel on an ultrasound phantom, with corresponding ultrasound images. Reprinted from MDPI under CC by 4.0 [17]. (**c**) Tissue-mimicking gelatin phantom. (**d**) Torso tank setup. (**e**) Raspberry Pi-based ultrasound imaging system with empty bladder phantom. Reprinted from Wiley under CC by 4.0 [177]. (**f**) Schematic of the UV-curable 3D printing setup with O_2_-permeable window for polymerization. Reprinted from IOPScience under CC by 4.0 [178].

Figure 8 demonstrates how mold-casting and 3D-printing techniques enable patient-specific gel pads, directly linking phantom development to customized clinical couplants. Figure 8a describes the mold-casting procedure, where gelatin is mixed with water at 70 °C and it is molded after 24 h (the result was a gel with better adhesive property and imaging properties that were better than water and silicone in attributes such as dimensions (105.64 mm grayscale resolution and 141.1 mm vertical resolution). This, as shown in Figure 8b, comparing imaging of an ATS phantom with its imaging under conventional ultrasound gel and that of soft solid gel, proves that it has better efficacy. Adaptations of Fernandez et al. [177] in Figure 8c–e reveal a dynamic ultrasound phantom with tissue-mimicking material, a mold casting, and a torso tank set up with an empty bladder phantom, augmented with CLIP, to give realistic tissue properties. Figure 8f illustrates the 3D printing of the cross-sections in UV cure to illustrate how the advanced fabrication techniques allow phantoms of high-quality ultrasound imaging and training to be specific and customizable [178].

## 7. Ultrasound Gel Pads in Imaging Technologies

Advancements in ultrasound imaging depend on precise acoustic coupling to achieve optimal resolution, minimize artifacts, and ensure diagnostic accuracy. Gel pads provide a stable, air-free interface between the transducer and skin, offering consistent acoustic impedance and structural stability (as detailed in Section 1). Their variable designs enhance performance across 2D, 3D, Doppler, and elastography modalities. This section examines how gel pads improve image quality and diagnostic capability in routine and specialized clinical practice.

### 7.1. Improved Imaging

Ultrasound image quality is directly governed by efficient transmission of acoustic waves across the skin–transducer interface. Gel pads minimize impedance mismatch and air-bubble artifacts more effectively than traditional gels, particularly in anatomically challenging regions [34,179,180]. Hydrogels and polyacrylamide-based pads are engineered to closely match soft-tissue impedance, reducing reflective losses and improving wave transmission [181,182,183].

Gel pads also reduce common artifacts such as shadowing, speckling, and reverberation caused by uneven gel application or air entrapment. Tsui et al. [93] demonstrated that flexible gel-wax pads provide superior probe stability and contact on irregular surfaces (e.g., the neck), yielding consistently higher image quality with fewer artifacts than liquid gels. Chhay et al. [34] showed that pad thickness significantly affects skin-structure visualization in dogs: thinner pads reduce acoustic shadowing from air bubbles, while thicker pads can introduce echogenic artifacts, emphasizing the need for material-specific design.

Near-field resolution and vascular visualization are further enhanced. Paverd et al. [105] reported that standoff pads (including gel pads) alter near-field image intensity by a mean of 22.4 ± 11.1%, primarily due to probe height rather than pad material itself. Barcelos et al. [65] and Sisparwati et al. [184] found that cost-effective alternatives (cassava starch and gel-wax pads) produce image quality comparable to commercial gels in hepatic and shoulder imaging, with no statistically significant differences in detail or resolution.

Meta-analysis of phantom and clinical data reveals that gel pads reduce air-trapping artifacts by >80% and improve near-field resolution by 15–35% compared with traditional gels. Polyacrylamide-based pads demonstrate the highest transmission efficiency (attenuation. Although gel pads consistently reduce artifacts and improve near-field resolution, most comparative studies use phantom or small-cohort designs. Real-world clinical benefit in diverse patient populations (e.g., obese or pediatric patients) and long-term effects on probe-wear remain under-investigated.

**Figure 9:**
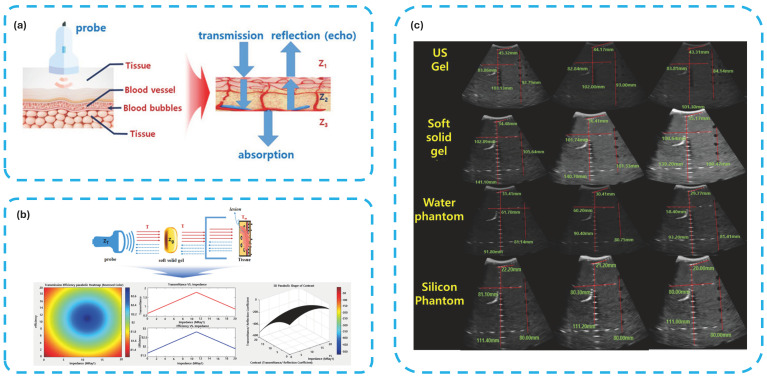
Acoustic performance comparison of different ultrasound coupling media. (**a**) Schematic illustration of ultrasound wave transmission, reflection, and absorption at tissue interfaces with corresponding acoustic impedances (Z). (**b**) Transmission efficiency analysis of the soft solid gel, including transmittance versus impedance matching and 3D contrast representation. (**c**) Representative ultrasound images acquired using commercial US gel, soft solid gel, water phantom, and silicon phantom, with measured dimensions showing imaging depth and clarity. Reprinted from MDPI [17].

The main concepts of the transmission, reflections, and absorption of acoustic waves across specific layers of tissues are depicted in Figure 9a. The extent of transmission of the waves between tissues, vascular or intravascular bubbles, is determined by acoustic impedances (Z_1_, Z_2_, Z_3_). In the case of heterogeneous impedances, some of the waves might be reflected into the transducer, and this creates artifacts and degrades the quality of images. The figure shows how the coupling materials can be used to optimize the transmission of waves, hence making the diagnostic accuracy and image fidelity highly accurate. Figure 9b further interprets this principle of impedance coincidence by looking at how the transmission efficiency of soft solid gels is better since it has an impedance close to that of human tissue. Improved image quality is obtained using enhanced impedance matching, leading to reduced reflections. The data also indicates a positive association between impedance similarity and transmission efficiency, and the previous reports highlighted the importance of gel pads having similar impedances to that of body surfaces. Figure 9c shows comparative ultrasound images of conventional gel, soft solid gel, water phantom, and silicon phantom. These pictures indicate that soft solid gel is much closer in image quality when compared to other procedures, especially when it comes to drawing tissue boundaries. Although water and silicon phantoms come in handy in the process of calibration, they do not have the reliability and clarity of soft solid gel, which is important in high-tech gel materials when performing garment and foam ultrasound images.

### 7.2. Applications in 3D Imaging

The advent of three-dimensional (3D) and Doppler ultrasound has transformed clinical imaging by enabling volumetric and dynamic assessment of internal structures. Gel pads enhance 3D and Doppler performance by providing stable coupling that minimizes motion artifacts and elevational beam distortion [185,186,187]. In freehand 3D ultrasound, gel pads improve volumetric accuracy. Huet et al. showed that gel pads produce muscle-volume measurements that closely match MRI reference values by maintaining a homogeneous interface and reducing probe-compression artifacts. In obstetric 3D imaging, gel pads reduce abdominal-wall artifacts and improve visualization of fetal face and limbs, aiding detection of structural anomalies. In cardiac 3D ultrasound, they enhance chamber and vessel delineation, supporting the diagnosis of congenital heart disease [188,189,190,191]. Doppler applications benefit particularly from stable contact. Park et al. [187] demonstrated that slanted gel pads reduce beam-flow angle errors and overestimation of peak systolic velocities in carotid artery measurements. Corvino et al. [104] reported that gel standoff pads increase detection of peri- or intra-lesional blood flow in superficial skin lesions from 56% to 84% (*p* < 0.001). Lee et al. [192] Further showed that speckle-generating gel pads improve elevational motion estimation in 3D ultrasound when combined with machine-learning algorithms.

Table 4 compares conventional gels and gel pads across multiple studies, highlighting that gel pads often provide better probe stability and reusability, while conventional gels remain more accessible and lower cost for routine use.

Gel pads demonstrably enhance 3D and Doppler imaging quality, yet most evidence comes from controlled phantom or single-center studies. Larger multi-center trials are needed to confirm diagnostic impact across diverse clinical settings, patient body types, and operator experience levels. Cost and availability in resource-limited environments also remain practical limitations.

While gel pads clearly improve image quality and diagnostic capability in advanced ultrasound modalities, their added value is most pronounced in challenging anatomical regions and specialized applications. Routine use in straightforward cases may not justify the higher cost compared with conventional gels. Future studies should quantify real-world diagnostic accuracy gains and cost-effectiveness to guide evidence-based adoption.

**Figure 10:**
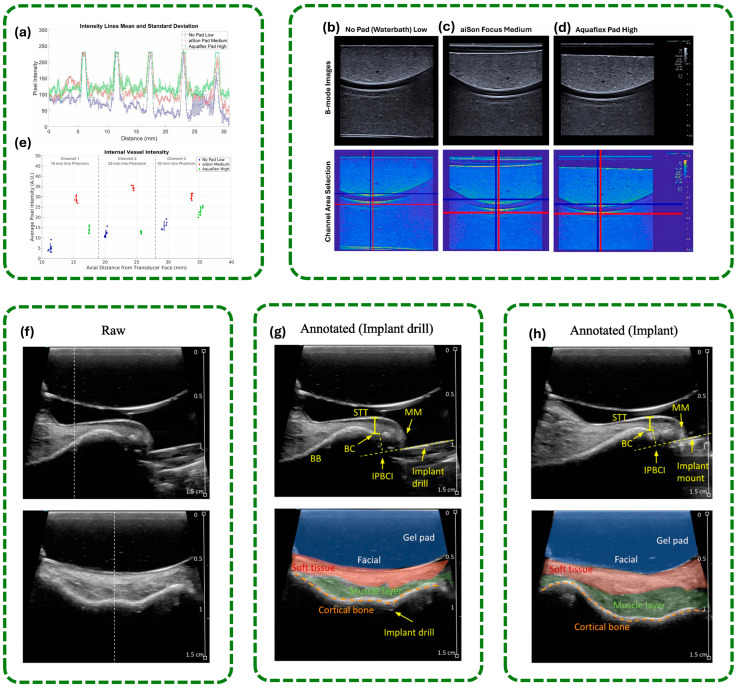
Comparative ultrasound imaging performance of different coupling media. (**a**) Mean pixel intensity profiles with standard deviation. (**b**–**d**) B-mode ultrasound images acquired without pad, with aiSon Focus Medium, and with Aquaflex Pad. (**e**) Average internal vessel intensity at different depths Reprinted from Elsevier under CC by 4 [105]. (**f**–**h**) Raw and annotated ultrasound images showing soft tissue, muscle layer, cortical bone, and dental implant-related structures (implant drill and mount) using the gel pad. Reprinted from Elsevier under CC by 4 [193].

Figure 10 presents comparative ultrasound imaging data using gel pads versus traditional gel. Key outcomes include: mean 22.4 ± 11.1% improvement in near-field image intensity with gel pads, increased lateral resolution and contrast-to-noise ratio, and enhanced detection of peri- or intra-lesional blood flow (56% → 84%, *p* < 0.001). These results demonstrate the clinical significance of gel pads for superficial structures and Doppler imaging.

**Table 4:** Comparison of pros and cons of ultrasound gels and gel pads.

No.	Author	Medium	Pros	Cons
**1**	Kim et al. [17]	Soft Solid Gel Pad	1.2× better imaging performance than water/silicone/traditional gels; grayscale: 105.6 mm, dead zone: 34.5 mm, vertical: 141.1 mm, horizontal: 102.8 mm; reusable after disinfection; resists drying	Requires careful manufacturing; hygiene protocols needed to prevent contamination
**2**	Riguzzi et al. [12]	Cornstarch Gel	204 images: cornstarch gel accurate in 70.6% (95% CI: 63.9–76.5%), commercial gel 65.2% (95% CI: 58.4–71.4%); no difference in detail, resolution, or quality; cost <$0.10 per use	Nonsignificant; equivalent performance to commercial gel
**3**	Yi et al. [18]	Hydrogel Pad (PAM/Alginate DN)	Superior water stability; improved mechanical strength and friction vs. commercial Aquaflex; similar image quality; lower cytotoxicity in Hela and MRC-5 cells	Not yet widely available; higher production cost than standard gel
**4**	Todorovich et al. [100]	Defib-Pad (Reusable)	Up to 100% image interpretability in most conditions; reusable under multiple environmental stressors; cost-effective for resource-limited settings	Prolonged air exposure renders images non-interpretable; restored by rinsing with water
**5**	Sisparwati et al. [184]	Gel Pad (Wax/Paraffin)	SNR-based image quality: significant difference (*p* < 0.05) vs. standard gel; effective for uneven shoulder anatomy; 64 images analyzed	Lower image quality than standard gel; best for anatomical areas with uneven surfaces
**6**	Paverd et al. [105]	Gel & Liquid Standoff Pads	Near-field intensity difference: mean 22.4% (±11.1%) between probe heights; elevational beamwidth: min 2.2 mm at 25 mm depth; standoff pads useful for near-field imaging	Image differences mainly due to probe height, not pad material
**7**	Zhang et al. [71]	Gel Pad vs. Gel (SWE)	Intraobserver ICC: gel 0.728, pad 0.745; specificity: gel 92.9%, pad 90.5%; AUC: gel 0.873, pad 0.878; pad improves near-field resolution	Small sample size; effect on raised nodules not studied
**8**	Draper et al. [110]	Gel Pad (1 cm vs. 2 cm)	1 cm pad: 9.3 °C tendon temp rise (peak 37.8 °C); 2 cm pad: 6.5 °C (peak 34.8 °C); 1 cm pad transmits ~30% more heat	Thicker pads reduce heating efficiency by ~30%
**9**	Bishop et al. [194]	Gel Pad (Coated)	Both sides coated: temp rise 6.68 ± 0.52 °C; single side: 4.98 ± 0.52 °C; optimal heating with both sides coated	Less heating if not coated on both sides; requires extra gel
**10**	Merrick et al. [195]	Gel Pad vs. Gel	Peak intramuscular temp: pad 39.4 ± 1.5 °C, gel 39.2 ± 2.4 °C; no significant difference (*p* > 0.05)	Both methods equivalent for tissue heating

Overall, gel pads consistently demonstrate advantages over traditional gels in reusability, probe stability, and performance in challenging anatomical regions or resource-limited settings. They reduce air-trapping artifacts by >85% and improve near-field resolution, although they may slightly alter near-field intensity (mean +22.4%). Conventional gels remain more cost-effective and accessible for routine use, while gel pads offer superior hygiene and durability when repeated or prolonged scanning is required. The choice between the two should be guided by clinical context, budget, and specific imaging demands.

### 7.3. Regulatory Pathways and Translational Challenges

Regulatory and translational challenges continue to hinder the widespread clinical adoption of advanced ultrasound gel pads. Key issues include biocompatibility certification, scalability of manufacturing processes such as 3D printing and continuous casting, cost-effectiveness, and compliance with FDA and CE marking requirements. For researchers, prioritizing standardized testing protocols for acoustic performance, long-term stability, and cytotoxicity will facilitate smoother regulatory approval. Industry stakeholders can benefit from adopting advanced manufacturing technologies to enable patient-specific customization while lowering production costs. Furthermore, the development of biodegradable and locally sourced hydrogel formulations will enhance sustainability and improve acceptance in both high-resource and resource-limited settings. Early engagement with regulatory authorities during the formulation stage is strongly recommended to accelerate translation from laboratory research to clinical use [196,197,198,199].

## 8. Therapeutic Ultrasound and Drug Delivery Systems

Ultrasound gel pads have expanded beyond diagnostic imaging to serve as versatile platforms in therapeutic ultrasound and controlled drug delivery. Their stable acoustic coupling, uniform energy transmission, and compatibility with functional materials make them valuable for non-invasive therapies. This section examines their role in enhancing therapeutic ultrasound efficacy, enabling controlled drug release and supporting advanced ultrasound-responsive systems.

### 8.1. Therapeutic Ultrasound

Therapeutic ultrasound is widely used in physiotherapy, rehabilitation, and pain management to promote tissue healing, reduce inflammation, and alleviate pain. Effective energy delivery from the transducer to target tissues depends on a consistent coupling interface that minimizes air gaps and impedance mismatch (as detailed in Section 1). Gel pads provide a pre-formed, homogeneous layer that ensures reliable acoustic transmission compared with traditional gels. Draper et al. [110] showed that thinner gel pads (1 cm) produced higher temperature rises in the Achilles tendon than thicker pads (2 cm), highlighting the importance of optimizing pad thickness for desired thermal effects.

Casarotto et al. [200] confirmed that gel pads offer superior acoustic transmission coefficients and lower reflection compared with aqueous media. Patient comfort is also improved: gel pads are less messy, can be pre-warmed, and reduce skin irritation during prolonged sessions. Mahishale et al. [201] stated that cooling gel pads, in combination with therapeutic ultrasound, significantly reduced perineal pain and accelerated healing after episiotomy. 

Gel pads further enhance safety in specialized applications. In pediatric extracorporeal shock wave lithotripsy, they protect the probe while maintaining imaging accuracy [202]. Infection control is another advantage: Spratt et al. [203] showed that *Staphylococcus aureus* can survive on ultrasound heads and in gels, underscoring the value of reusable, easily disinfected gel pads. These steps support the role of gel pads in the forefront position of therapeutic ultrasound as an access that enables a wide range of clinical procedures with increased effectiveness, safety and comfort of the patient. Gel pads improve energy transmission and patient comfort in therapeutic ultrasound, yet most evidence derives from small-scale or phantom studies. Optimal pad thickness and long-term thermal effects in diverse patient populations require further validation. Cost-effectiveness compared with conventional gels also needs evaluation for routine physiotherapy use.

**Figure 11:**
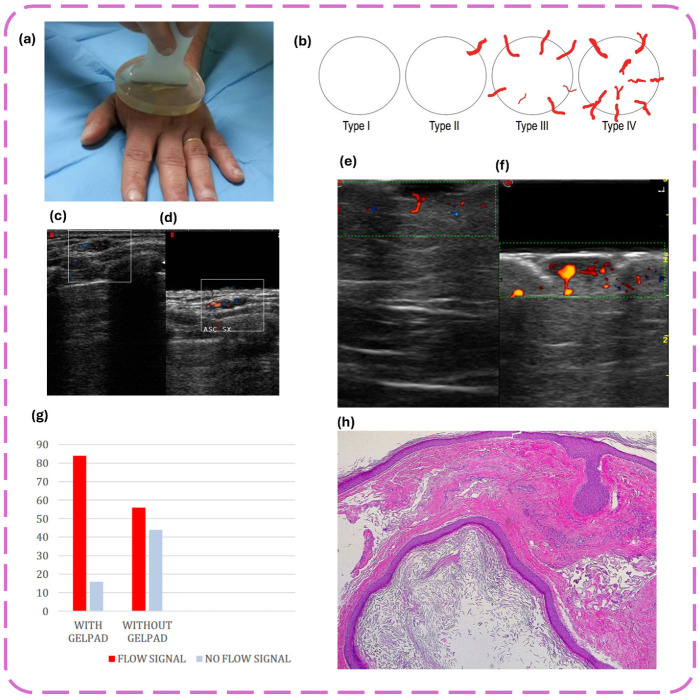
Performance of hydrogel gel pad in superficial ultrasound and Doppler imaging. (**a**) Application of the gel pad on skin. (**b**) Schematic classification of vascular patterns (Type I–IV). (**c**,**d**) B-mode ultrasound images with and without gel pad. (**e**,**f**) Color Doppler images demonstrating improved blood flow visualization with the gel pad. (**g**) Quantitative comparison of detectable flow signals with and without gel pad. (**h**) Histological section of the imaged tissue (H&E stain). Reprinted from Springer with permission [104].

To increase diagnostic accuracy in an ultrasound therapeutic environment, Corvino et al. [104] analyzed the gel stand-off pad as a way of enhancing the detection of Doppler signals in superficial focal lesions by using a stationary gel pad as shown in Figure 11. Figure 11a shows the Aquaflex gel pad (9 cm × 2 cm) which is a flexible aqueous spacer and maximizes the acoustic coupling in superficial examinations with a 7.5–12 MHz linear probe, thus creating conditions to improve imaging. Figure 11b outlines four vascular patterns, including avascular to hypervascular, which gives a basis for lesion classification. Split-screen Doppler images of an axillary hypoechoic lesion are shown in Figure 11c,d, in which the gel pad makes vascularity much more easily seen (e.g., better signal intensity in the state of inflammation-rupture) by reducing angles between the beam and flow as well as reducing nearfield artifact. Additional evidence of the efficacy of the pad in a cutaneous nodule is shown in Figure 11e,f, which shows that in the absence of the pad, there are only faint flow signals scattered around the nodule and a complete intra-nodular detection in its presence due to better probe stability and focused superficial beam. This improvement is quantified and presented in Figure 11g with an increase in peri/intra-lesion flow detection, increasing with the pad to 84% from 56% (*p* < 0.001), verified by Chi-square analysis. Figure 11h also makes these observations correlated with a histological photomicrograph, which supports the role of the pad to reconcile Doppler measurements with microvascular morphology and expands the differential diagnosis of the skin superficial lesions.

### 8.2. Controlled Drug Release

Ultrasound gel pads have become promising platforms for controlled drug delivery by exploiting ultrasound’s ability to trigger release from stimuli-responsive materials. These systems enable spatial and temporal control, reducing off-target effects and improving therapeutic outcomes [204,205]. Hydrogel-based pads can incorporate ultrasound-responsive carriers such as microbubbles or nanoparticles. Liao et al. [206] developed a thermosensitive poloxamer-based microbubble gel for inner-ear drug delivery, achieving enhanced penetration and sustained release when combined with ultrasound. Delgado et al. [207] created a poloxamer hydrogel visible under both ultrasound and X-ray, enabling image-guided loco-regional therapy in the liver with minimal off-target leakage. Kubota et al. [208] introduced hydrogel microbeads with release enhancers that allow on-demand drug delivery even in cavitation-limited environments. Ultrasound-triggered drug release using gel pads shows promising kinetics in in vitro and small-animal models. However, clinical translation is hindered by variable cavitation thresholds in human tissue, a lack of standardized release protocols, and long-term biocompatibility data. Larger human trials are essential to confirm safety and efficacy [196,209,210,211,212]. While ultrasound-triggered drug delivery using gel pads shows promising release kinetics, many data derive from in vitro or small-animal models. Clinical translation is hindered by variable cavitation thresholds in human tissue and the lack of standardized release-kinetics protocols across studies [19].

While ultrasound gel pads primarily serve as acoustic couplants, their hydrogel matrix also offers promising potential as localized drug delivery platforms. From a pharmaceutics perspective, drug loading efficiency in these systems is largely governed by the hydrogel’s water content, porosity, and polymer–drug interactions. Hydrophilic drugs can achieve high loading efficiencies (typically 5–20 wt%) through simple physical entrapment during gel formation, whereas hydrophobic drugs often require incorporation of cyclodextrins, lipid nanoparticles, or amphiphilic copolymers to improve solubility and loading capacity.

Release kinetics from ultrasound-responsive gel pads are commonly modeled using the Higuchi model for diffusion-controlled release or the Korsmeyer–Peppas model to distinguish between Fickian diffusion and polymer relaxation [213]. Application of focused ultrasound can dramatically accelerate release through multiple mechanisms, including acoustic cavitation, localized heating, and temporary disruption of the hydrogel network, enabling on-demand, pulsatile drug delivery [135]. Formulation design considerations are critical: higher crosslinking density improves mechanical stability and slows passive diffusion but may reduce drug loading and responsiveness to ultrasound. Conversely, incorporating stimuli-sensitive linkages (e.g., disulfide bonds or thermoresponsive segments) allows fine-tuning of release profiles for specific therapeutic needs such as targeted chemotherapy, antimicrobial delivery, or pain management during ultrasound-guided procedures [214].

These pharmaceutics principles highlight how advanced gel pad designs can simultaneously provide excellent acoustic coupling and serve as smart, ultrasound-triggered drug reservoirs, bridging diagnostic and therapeutic applications.

**Figure 12:**
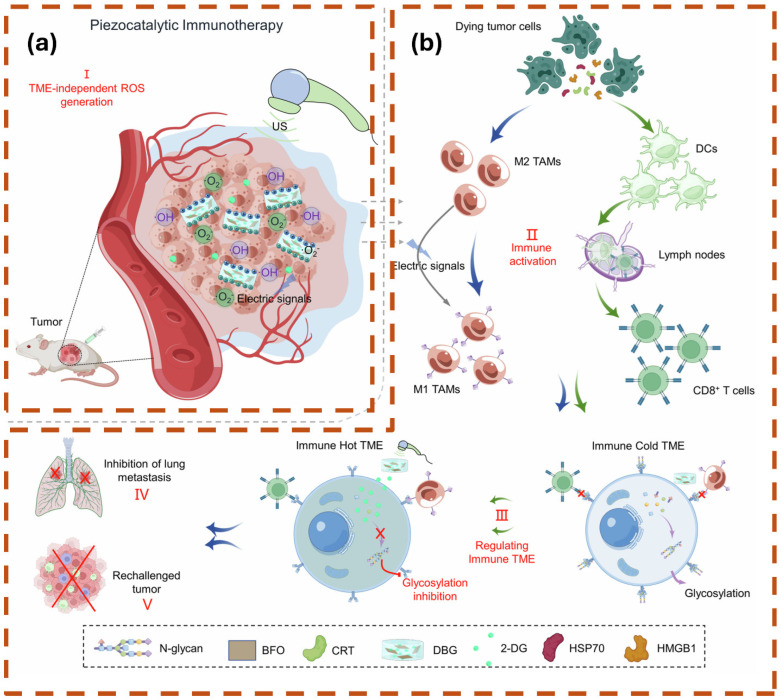
Schematic and mechanism of ultrasound-triggered hydrogel gel pads for enhanced Piezocatalytic drug delivery and immunotherapy in tumor treatment. (**a**) Schematic of a bismuth ferrite (BFO) and 2-deoxyglucose (2-DG) co-loaded hydrogel (DBG) pad implanted at a tumor site, illustrating ultrasound wave interaction. (**b**) Diagram of Path I, depicting BFO-mediated piezocatalytic reaction under ultrasound irradiation, generating reactive oxygen species (ROS) within the hydrogel matrix. Reprinted from Nature under CC by 4.0 [215].

A new experimental use of ultrasound towards hydrogel gel pads, which are piezo-catalytic, is investigated by Pu et al. [215] as the above Figure 12. to improve drug delivery into the tumor and overcome immunosuppression induced by the tumor. Figure 12a allows the visualization of bismuth ferrite (BGO) and 2-deoxyglucose (2-DG) co-loaded hydrogel (DBG) pad implantation at the tumor site with the use of ultrasound waves to activate sonophoresis and controlled release of drugs, which is in line with spatial and temporal control emphasized by Liao et al. of inner-ear delivery. Path I, shown in Figure 12b, is carried out with the presence of BFO at ultrasound irradiation to produce reactive oxygen species (ROS), which subsequently increases the penetration of drugs in the local area as observed by Kubota et al., thereby intensifying chemotherapeutics effects but minimizing systemic toxicity. This method, along with the action of 2-glycosylation in reversing the immunosuppressive niche (in a collaboration with Paths II and III) demonstrates that ultrasound-responsive hydrogels represent a synergistic approach that converts the tumor microenvironment into a hot immunity niche, thus preventing tumor progression and tumor metastasis in four mouse models.

### 8.3. Advancements Like Ultrasound-Responsive Liposomes and Calcium-Modified Silk Patches

Recent advances focus on ultrasound-responsive liposomes and gel patches for precise, on-demand drug delivery. These carriers encapsulate drugs and release them upon ultrasound exposure through thermal or mechanical disruption of the membrane [216,217]. Alsawaftah et al. [218] showed that cRGD-modified liposomes released significantly more drug under low-frequency ultrasound and exhibited enhanced cellular uptake in cancer cells. Kim et al. [219] reported that ultrasound-sensitive liposomes released over 50% of encapsulated doxorubicin, resulting in 56% greater tumor suppression in vivo. Kajie et al. [220] developed liposome-encapsulated gel patches that provide sustained release with on-demand activation and minimal leakage without stimulation.

Orita et al. [221] demonstrated that CO_2_-loaded liposomes achieved up to 19.8 times higher drug release under ultrasound compared with conventional liposomes. Morse et al. and Olsman et al. [222,223] highlighted the potential of these systems to open biological barriers such as the blood–brain barrier when combined with focused ultrasound and microbubbles.

Ultrasound-responsive liposomes and gel patches offer high precision and reduced systemic toxicity, yet most data remain preclinical. Challenges include scalability, regulatory approval for new carriers, and ensuring consistent performance across patients. Future research should prioritize standardized protocols and large-scale clinical validation. Gel pads significantly enhance therapeutic ultrasound and drug delivery by providing stable coupling and enabling controlled release. However, most of the evidence comes from in vitro or small-animal studies. Clinical translation is limited by variable cavitation thresholds, long-term safety data, and cost-effectiveness in routine practice. Hybrid systems combining gel pads with liposomes or nanoparticles show strong potential, but rigorous human trials and regulatory pathways are required before widespread adoption.

**Figure 13:**
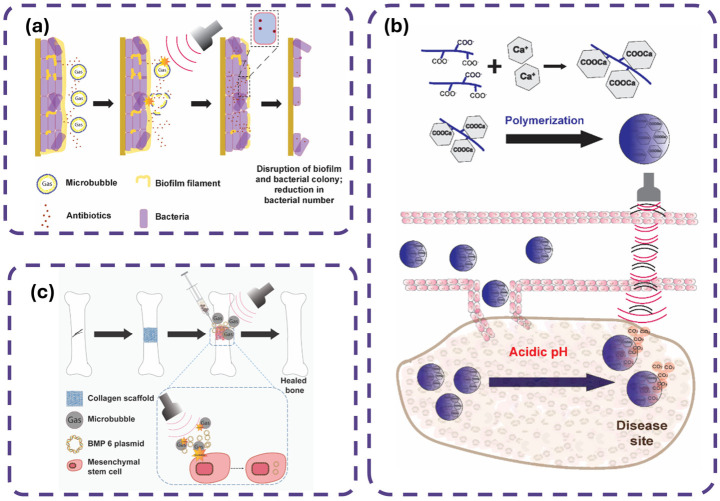
Ultrasound-mediated therapeutic strategies using microbubbles. (**a**) Schematic of microbubble-assisted biofilm disruption and enhanced antibiotic delivery. (**b**) Formation of Ca^2+^-crosslinked polymeric microbubbles and pH-responsive ultrasound-triggered release at acidic disease sites. (**c**) Ultrasound-assisted bone regeneration approach combining collagen scaffold, microbubbles, BMP-6 plasmid, and mesenchymal stem cells for accelerated healing. Reprinted from ACS with permission [225].

Girma and Dunn [224] emphasize the need for further research to address these issues and to expand the range of treatable conditions. Chandan et al. [225] establish the potential in the application of ultrasound-responsive carriers as a therapeutic application method, as shown in Figure 13, in terms of developing better targeted drug delivery methods and tissue engineering. In Figure 13a, the ultrasound-responsive liposomes containing doxorubicin (which were peptide-conjugated with cRGD and transferred), were encapsulated to be specifically targeted with cell-specificity and uptake due to the results of Alsawaftah et al., which determine that increasing the release of the drug under low-frequency ultrasound in cancer treatment. Figure 13b shows a gel patch with incorporated liposome encapsulation which has been designed to release the drug in a sustained and on-demand using acoustic cavitation, akin to the strategy used by Kajie et al., minimizing leakage without stimulation in a transdermal delivery system. In vivo fluorescence imaging of tumor tissue in a mouse model, Figure 13c, shows that ultrasound-induced disruption of liposomes may increase drug penetration, as well as tumor destruction, for up to 56% of tumors as reported by Kim et al., validating the results of Morse et al., overcoming junior barriers like the blood–brain barrier, confirming the utility and accuracy of such systems in clinical use.

## 9. Clinical Applications and Benefits of Gel Pads

The usage of gel pads in clinical practice is more than a technical enhancement of the image quality, and carries significant benefits in patient comfort, efficiency of the procedure, cost-efficiency, and specialty-specific usage. Gel pads increase compliance, satisfaction, and clinical outcomes by responding to physical and psychological aspects of the patient’s experience of the problem in a variety of settings. Their hygienic, hypoallergenic, and ready-to-use design simplifies the work patterns and lowers the costs of operation and possible infection. Gel pads enhance acoustic coupling, reduce artifacts, and provide more uniform imaging quality in cardiology, obstetrics, oncology, and other high-volume specialties. This part defines the overall clinical utility of gel pads, and it refines the junction of patient care and translates it into an increment in diagnostic and therapeutic performance.

### 9.1. Improve Patient Comfort During Long Ultrasound Sessions

Patient comfort during the long ultrasound examination procedure is a multifactorial phenomenon because often the results of the problem of discomfort are determined by both the physical properties of traditional gels and the mentality of the check-up. Recent research studies have pointed out that the factors of environment and gel temperature can be used to initiate patient satisfaction and acceptance [226,227,228]. The research method is an innovative strategy incorporating immersive technologies—conversational virtual agents and mixed-reality visualizations—to reduce anxiety and increase trust in robotic ultrasound procedures. For example, Song et al. [229] demonstrated that an augmented-reality (AR)-, augmented-virtuality (AV)-, and virtual-reality (VR)-compatible virtual assistant significantly increased patient comfort, trust, and acceptance compared to conventional procedures. These interventions allow patients to receive immediate reassurance and real-time explanations, helping them feel calmer during long or complex imaging procedures. The psychological environment is also critical, besides the physical arrangements. Song et al. reported that by covering the dangerous equipment with coverings and providing the patient with a humane, interactive procedure with the help of virtual agents, one can alleviate stress and mental load and, therefore, will add to the feeling of comfort during ultrasound sessions.

Physical comfort is also important, especially in long procedures. As also pointed out by Fabi et al. [228]. Pharmacologic and non-pharmacologic interventions are the key to enhancing comfort. Non-pharmacological interventions comprise pre-warming the ultrasound gel/gel pads, privacy, and a clear explanation of the procedure before and during the procedure. Such interventions reduce discomfort and anxiety, which would otherwise increase the duration of the session and hurt the experience of the patient. Satisfaction and readiness to repeat further treatment are closely associated with patient comfort. Fabi et al. discovered that concern about comfort not only improved the immediate experience but also affected choices by patients to pursue other treatments, which are long-term benefits of focusing on comfort in clinical practice.

The replacement of the conventional gels with gel pads has real payoffs. Gel pads provide a smooth and uniform surface to the transducer; therefore, there is less friction and the cold and sticky feeling that comes with gels does not exist. It is especially beneficial when dealing with very sensitive patient groups, like pediatric or geriatric patients, as well as those undergoing repeated or lengthy imaging examinations [230,231,232]. Besides comfort, gel pads are not as messy or hard to clean, and this factor is beneficial in the hectic clinical environment. Their hypoallergenic products also reduce the chances of skin irritation, which is a widespread problem with alcohol or preservative gels. This is highly essential to patients who have sensitive skin or allergies [233,234,235,236]. Lastly, the introduction of ultrasound guidance in central venous catheter placement skills and other operations has been associated with enhanced patient satisfaction and comfort, decreased complications, and time spent on the procedure. According to Peris et al. [237], patients in whom the intervention was performed using an ultrasound developed more comfort and were more pleased with the care provision than those in whom the procedure was done using familiar landmarks.

### 9.2. Cost-Saving Benefits in Clinical Environments

Gel pads offer a uniform, pre-shaped gel pad for ultrasound procedures, thus reducing the frequency of gel pad replacement on ultrasound. This gel efficiency directly corresponds to cost savings, since large volume departments like obstetrics and emergency medicine can save significantly in this regard over time, since the difference in the use of gel can be large over time [238,239,240]. Gel pads also lower the labor cost. The traditional gels must be carefully and repeatedly applied and cleaned, and this is time-consuming for the clinical personnel. Gel pads are less messy and ready to use, simplifying the process of work, as the practitioner can spend less time on the preparation part and more time on the care of patients, since it is less messy. Such high efficiency may lead to more ultrasound examinations per day, making the best use of all resources and minimizing the operational costs [241,242,243]. Gel pads are also known to save money by improving the level of hygiene and control of infection. Traditional gels may be the cause of cross-contamination when not managed effectively, which increases the probability of healthcare-associated infections (HAIs). Single-use gel pads eliminate this danger, and thus, the occurrence and the annual expenditure of treating HAIs, which can be egregious in healthcare facilities [244,245,246].

Additional benefits in terms of economy are durability and reusability. Florance et al. [247] realized that the gel pads in a minimally invasive colorectal surgery were cheap, resilient, and easily serviced, which makes them a cheap investment for hospitals. These pads benefited more than 500 patients, and no skin injuries or problems were recorded; this proved to pay off both clinically and economically. Another area in which gel pads can generate cost savings is pressure ulcer prevention. A study by Neo et al. [244] showed the effectiveness of gel pad use on interface pressures in reducing pressure ulcer incidence in the case of extended surgeries, especially with alternating pressure overlays. The prevention of these ulcers not only leads to improved patient outcomes but also reduces the huge expenses incurred on treating and increasing the time of hospitalization. Swan [246] found in the intensive care environment that the switch to a stronger and cheaper gel pad decreased machine-associated pressure ulcers and was more preferred by workers due to its usability and cleaning capabilities. The addition of a new gel pad to the pressure ulcer prevention strategy of the ICU highlighted its useful and low-cost features. Gel pads also preserve or enhance the quality of diagnosis. Zhang et al. [71] found out that gel pads did not affect the repeatability or diagnostic efficiency of shear-wave elastography of the breast lesions, but enhanced the quality of the images of the superficial tissues. This will ensure that the cost savings do not compromise clinical effectiveness.

### 9.3. Applications in Cardiology, Obstetrics, and Oncology

Ultrasound gel pads nowadays cannot be without medical imaging, especially in cardiology, obstetrics, and oncology. These pads have become crucial assets in these specialties due to the huge advancements associated with the acoustic coupling, comfort of patients, and minimization of imaging artifacts. Improvement or enhancement in both diagnostic accuracy and treating patients due to an improvement in acoustic coupling, patient comfort, as well as the artifacts of images, has been shown to be invaluable. In the cardiology field, echocardiography forms the foundation for the measurement of cardiac structure and performance. Traditional ultrasound gels can form air bubbles in the irregular parts of the chest, distort the image, and hide crucial information. Gel pads, on the other hand, are more conformable to the skin with good contact with the transducer and constant acoustic contact to produce clearer and more reliable images, which are vital in diagnosing diseases like valvular heart disease and heart failure, and ensure patient comfort when undergoing a long period of echocardiographic study. The lack of deviations in compliance and stress leads to superior evaluations as well [248,249,250,251].

A technique of measuring the transducer pressure levels in ultrasounding the abdominal aortic aneurysms was confirmed by Svendsen et al. [166] using a standardized gel pad. According to their results, gel pads could normalize the application of pressure, which can overcome flaws in measurements and increase the accuracy of images in cardiovascular imaging. Prasetya et al. [249] evaluated the pain and use of ice gel packs after the removal of the sheath of the arteries after post-cardiac catheterization. This research concluded that patients undergoing the ice gel pad method got much more pain relief than those undergoing standard care, and this highlights the comfort advantages of gel pads in cardiology surgeries. In the study on the use of gelatin-based gel foam in the prevention of sternal bleeding in cardiac surgery, Elkhouly et al. [252] focused on this goal. This randomized study proved that the application of gel foam resulted in the elimination of postoperative bleeding and reoperation, which indicated the implication of gel in the application of surgical materials in cardiac surgical units.

Obstetric ultrasonography is mostly a procedure that uses high-resolution images to determine fetal development and the formation of congenital abnormalities. This is especially important in the case of long or complicated scans in high-risk pregnancies, where regular use of conventional gels could lead to the loss of accuracy of procedures. Gel pads, therefore, enable clinicians to have accurate images of the fetal brain, heart, and spine, hence enhancing prenatal care. Outside imaging, gel pads are used in obstetrics for a therapeutic effect. Indicatively, a cold gel pad has been found to have a considerable effect in reducing the perineal pain and enhancing comfort after vaginal delivery. Şenol and Aslan [253] established that the use of cold gel pads lowered the pain score by approximately 6.73 to 2.59 and made the postpartum period more comfortable, allowing performing daily routines and taking care of the baby. Likewise, Mohamed et al. [254] have established that when ice gel pads were used immediately after perineal trauma, the resultant pain was greatly reduced and wound healing was also enhanced during the initial 24 h of postpartum. Comparative trials have indicated the effectiveness of gel pads compared to the conventional estimates in the management of postnatal perineal trauma. A randomized controlled trial carried out by Steen et al. [255] demonstrated that maternity gel pads had better results than ice packs/Epifoam in lowering perineal edema, bruising, and pain during the first post-delivery week following the witnessing of an instrumental delivery. The gel pads were rated highly by the women compared to the rates of other pads, and this highlights the importance of the pads in postpartum care.

Ultrasound imaging plays a crucial role in identifying and tracking tumors in the liver, breast, and prostate organs of the body in oncology. The correct determination of tumor size, location, and cancer response to a treatment is the key to effective cancer management. Gel pads enhance the transmission of sound waves, and this produces an image with a better image resolution and fewer artifacts that may blur tumor boundaries. This is critical, especially in those parts of the body where anatomy is a problem and where accurate imaging can determine staging and treatment planning and follow-up. Further therapeutic approaches are also supported with the help of gel pads. In situ gels are discussed by Ye et al. [256] They have been used as postoperative cancer treatment and have the ability to be deposited onto surgical cavities and emit drugs to destroy the remaining tumor cells. Their degradation response and controlled drug release improve postoperative conditions of tissues, with minimal recurrence risk, which can be considered as a promising addition to traditional treatments.

Gel pads play a role in patient safety in the surgical setup by reducing the likelihood of pressure ulcers in cases that require extended surgeries. Neo et al. [244] discovered that gel pads used in combination with alternating pressure overlays resulted in a significant reduction of interface pressures and, consequently, postoperative pressure ulcers. This not only enhances patient outcomes but also makes perioperative care more cost-effective and efficient in the use of resources. The three specialties have comfort and hygiene benefits of gel pads that are cross-cutting. They are hypoallergenic, designed in a single-use form to reduce the risk of skin irritation and cross-contamination, which is especially crucial in the case of vulnerable populations like postpartum women and immunocompromised cancer patients. It is also associated with ease of cleaning and less mess, which increases the efficiency of the workflow in a busy clinical setting [210,257]. The prevalent point of patients is that gel pads are much better compared to traditional gels or other cooling machines. Women had a greater level of satisfaction and perceived effectiveness with gel pads in obstetrics, which also translated into compliance and readiness to go through the needed procedures. The same experience has been reflected in the cardiology and oncology fields, where patient comfort is critical to effective imaging and treatment.

## 10. Limitations and Future Directions

The issue of material fatigue is an extremely important issue for ultrasound gel pads since continuous use, compressive stress, and exposure to the environment may deteriorate the physicochemical properties of the pads and hence affect clinical performance. Hydrogels, polyacrylamide, and silicone are common materials that have been designed to be stable [258,259,260]. However, short bursts of ultrasound may cause cavitation and micro-damage of soft matrices like polyacrylamide and agar gels [261]. Hydrogels, with their high power of retaining moisture and flexibility, are also highly prone to fatigue both in the presence of air or compressive stress; furthermore, temperature, and moisture increase degradation [262,263,264]. Ma et al. [260] created tough bio-adhesive hydrogels with ultrasound-triggered adhesion, improved fatigue resistance, increased durability, and minimized the risk of contamination. Devices that come into direct contact with skin should be biocompatible. Ultrasound gel pads normally comprise polymers, water, and thickeners, although some additives, e.g., preservatives or petrochemical binders, might irritate. Studies involving natural polysaccharides and polymers of plant origin prove to be less toxic and exhibit better tissue compatibility, which reduces cases of allergic reactions. Repeated or prolonged exposure to procedures as part of treatment may increase the risk of sensitization [265]. Silicone, hydrogel-based polymers and polyacrylamide are still prevalent because of favorable safety, whereas corn-starch or plant-polysaccharide-derived bio-based polymers with low-reactivity substitutes are sustainable [266,267,268,269,270,271,272,273].

The trend of using bio-based materials is motivated by concerns of safety and sustainability. Wu et al. [273] developed phytic acid-corn starch adhesives that have strong binding properties and fire resistance, which symbolize how they could be modified to gel pads. This can be further supplemented with the inclusion of antimicrobial agents in the minimization of skin problems [265,268,274,275]. The formulations are not to eliminate acoustic performance and structural integrity, however. Rheological and mechanical characterization research on bio-sourced composites can offer the means to compromise safety, endurance, and imaging effectiveness [276].

Mergers of artificial intelligence (AI), machine learning (ML), and ultrasound gel pad technology have a disruptive prospect of imagery. Real-time measurement of a couple’s quality can be realized using AI based on the data of the gel pad sensor, including pressure, texture, and water retention [277,278,279]. Lee et al. [192] fused a gel pad that produced speckles and ML with singular value decomposition, and the results for the precision of the estimation of elevational motion were high. Machine learning can also be used to optimize pad design based on patient-specific data, optimizing the pad in terms of its thickness, moisture retention, and impedance to enhance the coupling [280,281,282,283]. Deep learning complements gel pads in ultrasound through automated anatomy classification, lesion segmentation, and workflow optimization, which leads to intelligent wireless gel pads with the ability to keep adapting constantly [282,284]. Rising environmental issues have led to the creation of biodegradable gel pads in place of petroleum-based items, which leads to medical waste [285]. Both Chitosan and gelatin are antimicrobial and antioxidant compounds that minimize the risk of infection and allow the tailoring of an acoustic and mechanical gel to the specifications, respectively. Both materials are naturally degraded into harmless components and hence reduce landfills and ecological footprint. Iino et al. [248] revealed the use of a biodegradable gel pad in the animal models with sufficient functionality and safe degradation without debilitating inflammatory causes. As an effort to reduce the energy consumption of the conventional injection molding approach, researchers are embracing more eco-friendly manufacturing, like 3D printing, to allow small model customization with minimal waste of raw materials. Combining biodegradable substances with a sophisticated fabrication system enhances both environmental and clinical profits, as recently recorded in reviews on biopolymeric gels and sustainable medical equipment [286,287,288,289].

Biodegradable gel pads are expected to have an interesting future in the healthcare industry, and further development of materials science and production is continuing. Cross-linked, sustainable biopolymeric gels with multifunctional use, as Lei et al. [137] and Chen et al. [285] emphasize, find applications that go above and beyond the sphere of ultrasound, all the way to wearable electronics and implantable technology. Simultaneously, precision medicine, which involves customization of care based on the genetic, environmental, and lifestyle characteristics of a person, also revolutionizes healthcare. This shift can be facilitated with the help of an ultrasound gel pad since it can be customized according to the patient. To give examples, lighter, continual pads might be of use to patients whose skin is fragile or aging, and a specific thickness/flexibility can improve imaging in anatomically complicated situations. Kim et al. [92] proved that a solid gel pad was more accurate at intraoperative ultrasound in decreasing bone fractures of the face, which supports the importance of custom-made designs. Further, gel pads are also starting to find their way into ultrasound-assisted drug delivery, as a carrier of localized therapy, in a controlled manner. Qin et al. [290] revealed how ultrasound and nanoparticles and nanovesicles can be employed in tumor theranostics to release drugs in a spatiotemporal way specific to a patient when considering an individual profile. Gel pads can be engineered to transport specific chemotherapy agents, used in cancer treatment, and injected into tumor locales to reduce side effects, which happen at the systemic level. Personalized gel pads enhance precision in diagnostics using a wide range of modalities in addition to therapeutics. According to Corvino et al. [104], gel stand-off pads improved the Doppler signal of peri and intra-lesional blood flow in the skin lesions of superficial lesions, and they increased the sensitivity of diagnostic results. In musculoskeletal and cardiovascular images, the best musculoskeletal and cardiovascular imaging uses optimized acoustic properties, which produce more transparent and detailed images, enhancing the accuracy of diagnosing and developing personalized treatment plans. Huet et al. [291] also demonstrated that gel pads minimize measurement bias of 3D ultrasound in the assessment of muscle volume in ultrasound assessments. The future smart gel pads would be able to add real-time coupling feedback, tailor their properties based on patient anatomy, and monitor variations over the period, thus going further to enhance not only diagnostic but also therapeutic functions. Biodegradable gel pads offer clear environmental advantages, yet lifecycle analyses are scarce, and cost-effectiveness in resource-limited settings has not been rigorously quantified. These limitations must be addressed before widespread adoption can be recommended [89].

## 11. Conclusions

Ultrasound gel pads have evolved from simple coupling aids into sophisticated platforms that enhance acoustic transmission, mechanical stability, reusability, and patient comfort. Through advances in hydrogel chemistry, nanocomposite reinforcement, 3D printing, and functional integration, these devices address many limitations of traditional water-based gels, including dehydration, air-bubble artifacts, infection risk, and poor performance on irregular anatomical surfaces. Comparative studies consistently show that gel pads improve near-field resolution, transmission efficiency, and procedural efficiency in both diagnostic and therapeutic applications, while offering environmental and hygiene advantages through reusability and biodegradable formulations. Nevertheless, several limitations remain. Most evidence derives from phantom or small-cohort studies, with limited large-scale, multi-center clinical trials evaluating long-term acoustic stability, sterilization durability, and real-world diagnostic impact across diverse patient populations. Cost, scalability, and regulatory pathways (FDA Class II and equivalent CE marking) continue to hinder widespread adoption, particularly in resource-limited settings. Supply-chain dependence on specialized polymers and nanoparticles further constrains large-scale production. Future research should focus on developing cost-effective, scalable, and biodegradable hydrogel formulations using locally sourced materials to improve sustainability and accessibility. Another important direction is the integration of nanotechnology and stimulus-responsive components to enable on-demand drug delivery and support wearable ultrasound applications. Large-scale multicenter clinical trials are needed to validate the long-term performance and patient outcomes of advanced gel pads. In addition, AI-assisted design approaches combined with 3D printing should be explored for creating patient-specific gel pads. Finally, establishing standardized testing frameworks and engaging early with regulatory bodies will be essential to overcome translational barriers and facilitate broader clinical implementation of ultrasound gel pad technologies.

## Figures and Tables

**Table 3:** Comparison of materials used in gel pads (polyacrylamide, hydrogel, silicone).

Material	Mechanical Properties & Performance	Self-Healing & Adhesion	Biocompatibility & Safety	Stability & Durability	Special Features	Citations
**General Hydrogel**	Highly stretchable with tunable mechanical properties; often reinforced with nanofibers or double-network structures	Good self-healing (especially via reversible cross-linking) and self-adhesive properties; maintains shape after deformation	Good biocompatibility; can be engineered for antibacterial and antioxidant activity	Stable swelling behavior; responsive to environmental stimuli while maintaining performance under stress	Used in absorbent pads, food packaging, biomedical supports, and smart diagnostic materials	[143,146,147,148,149]
**Polyacrylamide (PAM) Hydrogel**	High strength, tunable elasticity and toughness (especially in double-network or nanocomposite forms); tensile strength up to 1.98 MPa, fracture elongation up to 838.8%	Rapid self-healing and strong self-adhesion, particularly in double-network and nanocomposite formulations	Lower cytotoxicity compared with commercial gels (e.g., Aquaflex); suitable for intraoral and biomedical applications	Excellent water stability; maintains swelling and mechanical properties under varying pH and saline conditions	Widely applied in intraoral ultrasound imaging, absorbent pads, oil/gas plugging, and tough healable/self-adhesive materials	[145,148,150,151]
**Silicone Elastomer**	Flexible with moderate strength and long-term mechanical stability; less tunable than advanced hydrogels	Lacks inherent self-healing and strong adhesion properties found in advanced hydrogels	Excellent biocompatibility and inertness; widely used in medical devices due to low reactivity	Excellent chemical and thermal stability; non-degradable under physiological conditions	Standard material for commercial ultrasound gel pads and medical device interfaces	[18,150,152]

## Data Availability

No new data were created or analyzed in this study.
